# Electrochemical and Surface-Enhanced Raman Spectroscopy-Based Biosensing Strategies for Wound Infection Detection: Recent Advances and Applications in Point-of-Care Diagnostics

**DOI:** 10.3390/bios16100567

**Published:** 2026-10-10

**Authors:** Rolla Mohamed, Abbas Sabahi, Somayeh Fardindoost, Mina Hoorfar

**Affiliations:** Department of Mechanical Engineering, University of Victoria, Victoria, BC V8P 5C2, Canada; rollae@uvic.ca (R.M.); abbassabahi@uvic.ca (A.S.); sfardindoost@uvic.ca (S.F.)

**Keywords:** wound infection, multimodal biosensing, electrochemical biosensors, surface-enhanced Raman spectroscopy (SERS), artificial intelligence

## Abstract

The growing prevalence of chronic wound infections presents a major global healthcare challenge due to delayed healing, prolonged hospitalization, and increased rates of limb amputation. Conventional diagnostic approaches, including clinical assessment, microbiological culture, and laboratory-based analyses, are often limited by subjective interpretation, delayed turnaround times, invasive sampling, and an inability to continuously monitor the dynamic wound microenvironment. Because wound infection involves changes in both host physiology and microbial activity, reliance on a single biomarker may not adequately characterize wound status, highlighting the potential of multimodal point-of-care diagnostic platforms. Recent advances in wearable biosensors, surface-enhanced Raman spectroscopy (SERS), nanomaterials, and artificial intelligence (AI) have supported the development of multimodal approaches for monitoring complementary host- and pathogen-derived biomarkers to facilitate rapid, non-invasive, and continuous wound infection assessment. This review examines selected multimodal biosensing approaches for wound infection detection, with a focus on electrochemical biosensors, SERS, and AI-assisted data analytics within point-of-care diagnostic frameworks. The review discusses (1) key wound infection biomarkers and the fundamental principles of electrochemical and SERS-based sensing; (2) recent developments in multimodal biosensing platforms, advanced plasmonic materials, and AI-assisted data interpretation and multimodal data fusion; and (3) current challenges and future opportunities for the clinical translation of point-of-care wound diagnostics. By combining complementary sensing modalities with AI-driven data analytics, these approaches may support earlier infection detection, continuous non-invasive monitoring, personalized wound management, and more informed clinical decision-making.

## 1. Introduction

### 1.1. Clinical Burden of Wound Infections

Wound infections remain a major healthcare challenge worldwide and are among the leading causes of delayed healing, prolonged hospitalization, limb amputation, and increased mortality [1]. Acute wounds typically progress through a coordinated sequence of hemostasis, inflammation, proliferation, and remodeling. However, disruption of this process by bacterial colonization, biofilm formation, impaired vascularization, diabetes, or immune dysfunction can result in chronic non-healing wounds [2]. Chronic wounds, including diabetic foot ulcers, pressure injuries, venous leg ulcers, and surgical site infections, affect millions of patients globally and represent a significant burden on healthcare systems. Among chronic wounds, diabetic foot ulcers are particularly concerning because individuals with diabetes often experience impaired circulation, peripheral neuropathy, and reduced immune function, all of which increase susceptibility to infection. Once infection is established, bacterial proliferation and biofilm formation can accelerate tissue destruction, increase the risk of osteomyelitis, and substantially raise the likelihood of lower-limb amputation. The prevalence of diabetes continues to increase worldwide, making infected diabetic wounds an increasingly important public health concern [2].

The economic burden associated with chronic wound management is substantial. Costs arise from frequent clinical visits, wound dressings, antimicrobial therapies, hospitalization, surgical interventions, and long-term care. In addition to direct healthcare expenditures, chronic wounds contribute to reduced quality of life, decreased mobility, loss of productivity, and significant psychological stress for patients and caregivers. Consequently, strategies that enable earlier infection detection and timely intervention have the potential to improve clinical outcomes while reducing healthcare costs [3]. One of the primary challenges in wound care is the delayed diagnosis of infection. Clinical assessment often relies on visible signs such as redness, swelling, pain, increased exudate, odor, and delayed healing. However, these symptoms may appear only after infection has progressed and may be difficult to interpret in patients with diabetes, vascular disease, or immunocompromised conditions. Furthermore, wounds often undergo dynamic biochemical and microbiological changes between clinical visits, making intermittent visual assessment insufficient for continuous monitoring. Early identification of infection-associated biomarkers before overt clinical symptoms emerge could significantly improve treatment decisions and prevent disease progression [4]. For these reasons, there is growing interest in developing advanced diagnostic technologies capable of continuously monitoring wound status and providing rapid, objective, and quantitative information regarding infection development. Such approaches have the potential to support personalized wound management and facilitate earlier therapeutic intervention [5].

### 1.2. Current Diagnostic Approaches

Current methods for diagnosing wound infection include clinical examination, microbiological analysis, molecular techniques, chemical analysis of wound metabolites, and medical imaging. Although these approaches provide valuable information, each presents important limitations that restrict widespread implementation for real-time point-of-care monitoring [4,6]. Visual assessment remains the most commonly used diagnostic approach in clinical practice. Healthcare providers evaluate wound appearance, tissue condition, exudate characteristics, odor, and signs of inflammation to determine infection status. While simple and inexpensive, visual assessment is inherently subjective and may vary considerably among clinicians. In addition, visible symptoms often appear only after infection has become established, limiting the ability to detect early pathological changes [7].

Microbiological culture obtained through wound swabs or tissue biopsies is widely regarded as the clinical reference method for identifying bacterial pathogens. Culture-based analysis enables species identification and antimicrobial susceptibility testing, thereby guiding treatment decisions. However, culture procedures typically require 24–72 h to produce results, delaying therapeutic intervention. Furthermore, culture techniques may fail to accurately represent polymicrobial wound communities and often underestimate the presence of biofilm-associated bacteria [8]. Molecular diagnostic techniques, including polymerase chain reaction (PCR), have improved sensitivity and specificity for pathogen identification. PCR-based methods can rapidly detect bacterial DNA and identify antibiotic resistance genes, often within hours. Despite these advantages, PCR requires specialized laboratory infrastructure, trained personnel, and relatively expensive instrumentation. Additionally, the presence of bacterial DNA does not necessarily indicate active infection, as genetic material may persist after bacterial death [9]. An important consideration when interpreting wound-infection biosensor studies is the reference standard used to define infection. In clinical practice, wound infection is primarily assessed using a combination of clinical signs and symptoms, including increasing pain, erythema, edema, purulent or abnormal exudate, malodor, wound deterioration, delayed healing, and other local or systemic indicators. International consensus documents emphasize that no single clinical sign is sufficient to define infection in all wound types, and that infection assessment should consider the overall clinical context and wound type [10]. Microbiological assessment is frequently used as an additional reference, with wound-tissue culture generally providing a more representative assessment of microbial burden than superficial swabbing; the Levine swab technique is commonly considered an acceptable clinical alternative when tissue sampling is impractical [9]. However, the reference standard is not consistent across biosensor studies. Some studies use qualitative or quantitative bacterial culture as the reference, whereas others use PCR-based pathogen identification, clinical diagnosis, bacterial load, or a combination of clinical and microbiological criteria [11,12]. Therefore, the diagnostic performance of individual biomarkers and wearable sensors should be interpreted in relation to the specific reference standard adopted in each study rather than assuming that all studies define infection using the same criterion.

More recently, volatile organic compounds (VOCs) have emerged as promising non-invasive biomarkers of wound infection. VOCs are chemically diverse carbon-containing molecules generated by microbial metabolism, host metabolism, tissue degradation, and interactions between microorganisms and the wound microenvironment. Unlike non-volatile biomarkers that are predominantly measured in wound exudate, VOCs can be sampled from the wound headspace, potentially enabling non-contact or minimally invasive monitoring. Bacterial species can generate characteristic volatile profiles through metabolic pathways involving amino acids, fatty acids, carbohydrates, and other substrates, thereby producing patterns that may distinguish infected from non-infected wounds or provide information about the microbial community [13].

The VOCs reported in association with wounds span several chemical classes, including alcohols, aldehydes, ketones, esters, hydrocarbons, sulfur-containing compounds, nitrogen-containing compounds, and short-chain fatty acids. Examples reported in wound-related studies include alcohols and ketones associated with microbial metabolism, aldehydes and hydrocarbons arising from metabolic and oxidative processes, and sulfur-containing compounds such as dimethyl trisulfide that contribute strongly to wound malodor. Short-chain fatty acids, alcohols, ketones, aldehydes, and sulfur-containing compounds have all been implicated in the characteristic odor of chronic wounds [13]. Importantly, individual VOCs are not necessarily specific to a single bacterial species; therefore, diagnostic approaches increasingly focus on characteristic VOC patterns or combinations rather than relying on a single compound.

The relationship between VOC concentration and wound infection should nevertheless be interpreted carefully. Increased abundance of a particular VOC may reflect bacterial metabolic activity, tissue necrosis, inflammation, or changes in the wound microenvironment rather than infection severity alone. Therefore, future VOC-based wound-monitoring systems should establish quantitative concentration ranges and validate VOC signatures against clearly defined clinical and/or microbiological reference standards. Combining multiple VOCs with exudate-based biomarkers may ultimately provide greater diagnostic specificity than VOC measurements alone [13].

Wound exudate is the principal biological matrix for many biochemical wound-monitoring approaches and provides a direct representation of the wound microenvironment. It is a complex fluid generated through increased vascular permeability during inflammation and contains water, electrolytes, proteins, albumin, glucose, lactate, urea, uric acid, inflammatory cytokines, growth factors, proteases, protease inhibitors, extracellular-matrix components, cellular debris, and microbial products. The composition of exudate changes dynamically with wound type, inflammatory status, bacterial burden, and healing stage. Consequently, measurements obtained from wound exudate should not be interpreted in the same manner as measurements obtained from blood or other physiological fluids.

Several studies have demonstrated clinically relevant quantitative changes in exudate biomarkers during impaired healing and infection. For example, wound-fluid lactate has been reported at a median concentration of approximately 21 mM (range 5.6–80.4 mM) in diabetic foot ulcers, with significantly higher levels observed in infected than non-infected wounds [14]. However, absolute biomarker concentrations should be interpreted cautiously because reported values vary substantially according to wound type, sampling method, exudate volume, normalization strategy, analytical technique, and disease stage. In particular, there is currently no universally accepted concentration threshold that independently defines wound infection or provides a standardized quantitative scale of infection severity for most biochemical biomarkers. Therefore, rather than assigning universal infection cut-offs, biosensor studies should report the measured concentration range, the wound population, the reference standard used for infection classification, and the observed relationship between biomarker concentration and clinical outcome.

### 1.3. Need for Multimodal Point-of-Care Diagnostics

The complex and dynamic nature of wound healing presents a significant challenge for infection diagnosis because no single biomarker can comprehensively describe wound status. The major biomarker groups relevant to wound monitoring can be broadly classified into physicochemical, metabolic, inflammatory, proteolytic, microbial, and volatile biomarkers. Their diagnostic significance depends not only on whether their concentrations increase or decrease during infection, but also on their quantitative range and relationship with wound progression. Physicochemical parameters such as pH and temperature provide relatively broad indicators of the wound microenvironment. Wound pH generally shifts from a more alkaline environment toward mildly acidic conditions during successful healing, whereas persistently elevated pH has been associated with chronic or non-healing wounds. Temperature may also increase in infected or inflamed wounds, although elevated temperature is not specific to infection and may also occur during normal inflammatory and reparative processes.

For example, elevated wound pH may indicate infection but can also result from normal inflammatory responses. Similarly, increased temperature may reflect infection, tissue repair, or mechanical irritation. Metabolic biomarkers such as lactate and uric acid provide important information regarding tissue hypoxia and bacterial activity but are insufficient when interpreted independently. Likewise, VOC analysis can reveal pathogen-specific metabolic signatures but may not adequately capture host inflammatory responses. These limitations highlight the need for multimodal sensing strategies capable of integrating complementary information from multiple biomarkers simultaneously [13,15]. Continuous monitoring represents another critical requirement for next-generation wound diagnostics. Traditional episodic measurements provide only isolated snapshots of wound status and may miss important temporal changes occurring between clinical evaluations. Continuous sensing platforms embedded within wound dressings can provide real-time information regarding infection progression, treatment response, and healing trajectories without requiring frequent dressing removal. Such approaches minimize wound disturbance while improving clinical decision-making.

Recent advances in wearable electronics, flexible materials, nanotechnology, and biosensor engineering have enabled the development of smart wound dressings capable of monitoring multiple biomarkers directly within the wound microenvironment [16]. Electrochemical biosensors have demonstrated considerable success for detecting pH, lactate, uric acid, glucose, and inflammatory markers in wound exudate [17]. Simultaneously, optical sensing technologies such as surface-enhanced Raman spectroscopy (SERS) have emerged as powerful tools for detecting bacterial metabolites and VOC fingerprints with exceptional molecular specificity and sensitivity [18,19]. The integration of artificial intelligence (AI) and machine learning further expands the potential of multimodal wound diagnostics. AI algorithms can analyze large multidimensional datasets generated by wearable sensors, identify complex biomarker relationships, recognize infection patterns, and support automated clinical decision-making. Machine learning approaches such as principal component analysis (PCA), support vector machines (SVM), random forests, and convolutional neural networks (CNNs) have already demonstrated promising performance for bacterial classification using electrochemical and SERS-derived datasets [20,21].

Collectively, these advances are driving a transition from conventional wound assessment toward intelligent multimodal diagnostic platforms that combine biochemical sensing, molecular fingerprinting, wireless communication, and AI-assisted analysis. Such systems have the potential to provide earlier infection detection, personalized treatment guidance, and improved clinical outcomes while reducing healthcare costs and patient burden. Following bacterial contamination, wounds progress through colonization, biofilm formation, local infection, and potential chronic wound development. Conventional diagnostic approaches, including visual assessment, microbiological culture, PCR, gas chromatography-mass spectroscopy (GC-MS) VOC analysis, and imaging techniques, provide valuable information but suffer from limitations related to sensitivity, turnaround time, cost, or accessibility. Emerging multimodal point-of-care platforms integrate electrochemical biosensors, SERS-based molecular fingerprinting, wearable wound dressings, wireless communication, and artificial intelligence to enable continuous and non-invasive wound infection monitoring. Figure 1 shows wound infection progression and emerging diagnostic technologies in three sections.

## 2. Biomarkers Associated with Wound Infection

Wound healing is a highly coordinated biological process involving interactions among immune cells, tissue structures, microorganisms, and biochemical signaling pathways. During infection, significant alterations occur within the wound microenvironment, resulting in measurable changes in physicochemical, metabolic, inflammatory, and microbial biomarkers. Monitoring these biomarkers provides valuable insight into wound status and enables early identification of infection before the appearance of overt clinical symptoms. Because wound infection is a multifactorial process, no single biomarker can accurately capture the complexity of disease progression. Instead, combinations of biomarkers reflecting host response, tissue metabolism, bacterial activity, and microbial metabolites offer a more comprehensive assessment of wound health. Recent advances in biosensing technologies have facilitated the simultaneous detection of multiple biomarkers, supporting the development of multimodal diagnostic platforms for wound monitoring [15,22].

### 2.1. Physicochemical Biomarkers

#### 2.1.1. pH

Wound pH is one of the most widely investigated indicators of wound healing and infection. Healthy skin typically exhibits a slightly acidic pH ranging from 4.5 to 6.0, which supports antimicrobial defense and normal tissue function. Following injury, wound pH often becomes more alkaline due to tissue damage, inflammatory responses, and bacterial colonization. Acute healing wounds generally exhibit pH values between 5.5 and 7.0, whereas chronic and infected wounds frequently display alkaline conditions with pH values exceeding 7.5 and sometimes approaching 9.0. Elevated pH promotes bacterial proliferation, increases protease activity, and impairs tissue regeneration. Consequently, continuous monitoring of wound pH has become an important strategy for assessing infection risk and healing progression [23].

#### 2.1.2. Temperature

Temperature changes provide valuable information regarding local inflammatory activity and infection development. Increased bacterial burden and immune activation stimulate blood flow and metabolic activity, resulting in localized temperature elevation. Clinical studies have demonstrated that infected wounds often exhibit temperatures several degrees higher than surrounding healthy tissue [24,25]. Temperature monitoring offers a non-invasive and easily measurable parameter for wound assessment. However, temperature alone lacks specificity because increases may also arise from normal healing responses, mechanical irritation, or systemic conditions. Therefore, temperature measurements are often combined with additional biomarkers to improve diagnostic accuracy [26].

#### 2.1.3. Oxygen

Oxygen plays a critical role in tissue repair, collagen synthesis, angiogenesis, and immune function. Adequate oxygen availability supports efficient wound healing, whereas hypoxia contributes to delayed tissue regeneration and increased susceptibility to infection. Chronic wounds frequently exhibit reduced oxygen availability because of impaired vascularization, edema, and increased metabolic demand. The partial pressure of oxygen (pO_2_) is approximately 45–65 mmHg at the normal skin surface but may decrease to 0–5 mmHg in the non-vascularized central region of a wound; pO_2_ values of 5–20 mmHg are commonly observed in non-healing wounds, whereas values of approximately 30 mmHg have been reported in infected or traumatized wounds [27]. Furthermore, bacterial proliferation and biofilm formation can further deplete local oxygen concentrations. Monitoring tissue oxygenation can therefore provide valuable information regarding wound viability, healing potential, and infection progression [27].

### 2.2. Metabolic Biomarkers

#### 2.2.1. Lactate

Lactate is a key metabolic product generated during anaerobic glycolysis and serves as an indicator of tissue oxygenation and cellular metabolism. Under normal physiological conditions, moderate lactate production supports angiogenesis and tissue repair. Excessive lactate accumulation is often associated with hypoxia, inflammation, and infection. In diabetic foot ulcers, the median wound-fluid lactate concentration was significantly higher in clinically infected wounds than in non-infected wounds: 27.18 mM (range: 7.14–80.40 mM) versus 18.38 mM (range: 5.58–50.34 mM), respectively (*p* = 0.001) [14]. These findings support the potential use of lactate as a biomarker for wound infection monitoring; however, the overlapping concentration ranges indicate that lactate should not be interpreted as an infection-specific marker when used alone [14].

#### 2.2.2. Glucose

Glucose plays a fundamental role in cellular metabolism and wound repair. In diabetic patients, dysregulated glucose metabolism significantly impairs immune function, collagen synthesis, and angiogenesis, increasing the risk of infection and chronic wound formation. Local wound-fluid glucose may decrease during infection because of bacterial consumption [28]. In a study diabetic porcine wound model, glucose in non-inoculated wounds decreased from 350 mg dL^−1^ (approximately 19.4 mM) on day 1 to 79 mg dL^−1^ (approximately 4.4 mM) on day 8 and became undetectable by day 12, whereas no detectable glucose was observed in wounds inoculated with *Staphylococcus aureus* [29]. Consequently, glucose monitoring has gained increasing attention, particularly for diabetic wound management, where metabolic control directly influences healing outcomes [28].

#### 2.2.3. Uric Acid

Uric acid is a product of purine metabolism and serves as a marker of cellular damage, oxidative stress, and inflammatory activity. In chronic venous leg ulcer fluid, uric acid accounted for approximately 65% of the total measured purines in more severe wounds, defined by Pressure Ulcer Scale for Healing (PUSH) scores of 10–16, compared with approximately 33% in less severe wounds with PUSH scores of 3–9 (*p* < 0.001) [30]. This association indicates that the relative uric acid level may provide information about wound severity. However, patients with clinical signs of infection were excluded from that study; therefore, these values should not be regarded as infection-specific concentrations or diagnostic thresholds [30].

### 2.3. Inflammatory Biomarkers

#### 2.3.1. Reactive Oxygen Species (ROS) and Hydrogen Peroxide (H_2_O_2_)

Reactive oxygen species (ROS), including hydrogen peroxide (H_2_O_2_), superoxide anions, and hydroxyl radicals, are generated by activated immune cells as part of the host defense response against invading pathogens. At controlled levels, ROS contribute to microbial elimination and cellular signaling during wound healing, whereas excessive and sustained ROS production promotes oxidative stress, cellular damage, and impaired tissue regeneration. Quantitative interpretation is challenging because ROS comprise chemically distinct and short-lived species, and no universally accepted ROS or H_2_O_2_ concentration range has been established for infected wounds. Experimentally, H_2_O_2_ concentrations of 25–50 μM produced bacteriostatic effects against *Escherichia coli*, whereas concentrations above 500 μM were required for bacterial eradication [31]. These values represent experimentally determined antimicrobial concentrations rather than H_2_O_2_ levels measured in infected wound exudate. Consequently, wound ROS measurements should be interpreted according to the detected species, analytical method, and biological context [31].

#### 2.3.2. Cytokines

Cytokines are signaling molecules that regulate immune responses and coordinate wound healing processes. Pro-inflammatory cytokines such as tumor necrosis factor-alpha (TNF-α), interleukin-6 (IL-6), and interleukin-1 beta (IL-1β) are often significantly elevated during wound infection. Persistent overexpression of these cytokines contributes to prolonged inflammation, delayed healing, and tissue destruction. Monitoring cytokine profiles therefore provides important information regarding immune activation and infection progression [32,33].

#### 2.3.3. Proteases

Proteolytic enzymes play essential roles in extracellular matrix remodeling and tissue repair. During infection, excessive protease activity can disrupt normal healing processes by degrading growth factors, extracellular matrix proteins, and newly formed tissue. Among these enzymes, matrix metalloproteinases (MMPs) are particularly important because elevated levels are strongly associated with chronic non-healing wounds. Consequently, protease monitoring has become a promising strategy for evaluating wound status and predicting healing outcomes [34].

### 2.4. Bacterial Biomarkers

Pyocyanin is a redox-active virulence factor produced almost exclusively by *Pseudomonas aeruginosa*, one of the most common pathogens associated with chronic wound infections. The molecule contributes to oxidative stress, tissue damage, and biofilm formation while interfering with host immune responses. Due to its electrochemical activity and high pathogen specificity, pyocyanin has become an attractive target for rapid electrochemical detection of *P. aeruginosa* infections. Many pathogenic bacteria communicate through quorum sensing systems that regulate collective behaviors, including virulence factor production, biofilm formation, and antibiotic resistance. Examples of quorum-sensing molecules relevant to wound-associated bacteria include N-acyl homoserine lactones (AHLs), such as N-(3-oxododecanoyl)-L-homoserine lactone (3-oxo-C12-HSL) produced by *P. aeruginosa*, as well as the Pseudomonas quinolone signal (PQS). Their concentrations can increase as bacterial populations grow and quorum-sensing activity becomes established within the infected wound environment. Monitoring quorum-sensing molecules can provide information on bacterial colonization and virulence-associated activity before overt clinical signs of infection develop, offering opportunities for timely clinical intervention [35]. Biofilms are highly organized microbial communities embedded within a protective extracellular polymeric matrix. Biofilm formation represents a major challenge in chronic wound management because biofilm-associated bacteria exhibit enhanced resistance to antibiotics and immune clearance. The presence of biofilm-associated biomarkers, including extracellular DNA, polysaccharides, proteins, and metabolic products, provides valuable information regarding infection severity and treatment resistance. Detection of these indicators has therefore become an important focus of advanced wound diagnostic technologies.

### 2.5. Volatile Organic Compounds

Volatile organic compounds are gaseous metabolites released during bacterial growth and metabolism. Increasing evidence suggests that VOCs can serve as non-invasive biomarkers for wound infection because they reflect real-time microbial activity and can diffuse from the wound environment without requiring direct tissue sampling. Different bacterial species produce distinct VOC mixtures that arise from specific metabolic pathways. Common wound-associated VOCs include alcohols, ketones, aldehydes, sulfur-containing compounds, fatty acid derivatives, and aromatic metabolites. These compounds collectively generate unique chemical fingerprints that can be used to identify bacterial presence and activity [20].

Several clinically relevant wound pathogens produce characteristic VOC signatures. For example, *Pseudomonas aeruginosa* generates compounds such as 2-aminoacetophenone and various sulfur-containing metabolites [36,37], while *Staphylococcus aureus* produces distinct aldehydes and ketones [38]. These species-specific profiles provide opportunities for pathogen discrimination without requiring culture-based methods. Unlike many conventional biomarkers that require direct contact with wound exudate, VOCs can be sampled from the wound headspace, enabling completely non-invasive monitoring. Combined with highly sensitive analytical techniques such as GC-MS and SERS, VOC analysis has emerged as a promising approach for rapid pathogen identification and early infection detection [39]. The growing recognition of VOCs as indicators of microbial metabolism has stimulated significant interest in wearable sensing platforms capable of continuously monitoring wound-emitted gases. Such technologies may complement liquid-phase biomarker measurements and contribute to the development of next-generation multimodal wound diagnostic systems.

## 3. Electrochemical Biosensors for Wound Infection Detection: Fundamental Principles, Biomarkers, and Transduction-Based Classification

### 3.1. Fundamental Principles of Electrochemical Biosensors

Electrochemical biosensors are analytical platforms in which a biological, biochemical, or physicochemical recognition event is converted into a measurable electrical signal through an electrode-based transducer, making them highly suitable for monitoring the dynamic wound microenvironment in real time [40]. In a typical configuration, the biosensor is composed of a recognition layer or bioreceptor, an electrochemical transducer, an electrode system, and a signal-processing/readout unit that converts the primary electrochemical response into clinically interpretable data [41]. The recognition element may be an enzyme, antibody, aptamer, nucleic acid, molecularly imprinted polymer, redox-active polymer, catalytic nanomaterial, or responsive hydrogel, depending on whether the target is a metabolite, bacterial product, inflammatory mediator, enzyme, pathogen, or physicochemical parameter [42]. The electrode system usually includes a working electrode at which the sensing reaction occurs, a reference electrode that stabilizes the potential, and a counter electrode that completes the circuit in current-based measurements [43]. The electrode system must be connected to appropriate electrochemical instrumentation to apply the required electrical excitation and acquire the resulting electrical response. Potentiostatic instrumentation is commonly used for amperometric and voltammetric measurements, whereas impedance-based measurements require an impedance analyzer or a potentiostat equipped with electrochemical impedance spectroscopy capabilities [43,44,45]. The acquired electrochemical signals may subsequently be conditioned, digitized, wirelessly transmitted through technologies such as Bluetooth or Near Field Communication (NFC), and processed or interpreted using smartphone-based or AI-assisted systems for wearable point-of-care wound monitoring [44,46,47].

The signal is generated when a wound-related analyte changes the interfacial electron-transfer process, ion activity, redox current, impedance, capacitance, conductivity, or open-circuit potential at the sensing interface [43]. In enzyme-based amperometric biosensors, the enzymatic conversion of glucose, lactate, uric acid, or oxygen-related substrates produces or consumes electroactive species such as H_2_O_2_, oxygen, or redox mediators, and the measured current becomes proportional to biomarker concentration [43]. In potentiometric sensors, wound pH or ion concentration is detected through a potential difference between a sensing electrode and a reference electrode under near-zero-current conditions, with the response usually being governed by ion activity and Nernst-type behavior [48]. In impedimetric biosensors, biomarker binding, bacterial attachment, biofilm growth, hydrogel degradation, or changes in wound exudate composition alter charge-transfer resistance, double-layer capacitance, phase angle, or bulk resistance across a frequency range [49]. In conductometric and resistive systems, variations in ionic strength, moisture content, protonation state of conducting polymers, or percolation of carbon-based networks cause measurable changes in conductivity or resistance that can be related to wound hydration, strain, temperature, or biochemical activity [42]. These signal-transduction mechanisms are particularly relevant to wound infection detection because wound infection is accompanied by rapid and heterogeneous changes in pH, temperature, uric acid, lactate, glucose, ROS/H_2_O_2_, cytokines, proteases, pyocyanin, nitric oxide, and bacterial burden that are difficult to detect through intermittent visual inspection or delayed microbiological assays [43].

### 3.2. Detection Targets in Wound Infection Monitoring

Electrochemical biosensors for wound infection monitoring have mainly been developed for pH, lactate, uric acid, glucose, H_2_O_2_/ROS, temperature-associated electrical readouts, oxygen, nitric oxide, pyocyanin, cytokines, proteases, and bacterial cells, although pH and uric acid have been the most frequently investigated wearable targets [43]. Wound pH is important because normal skin is acidic, whereas infected and chronic wounds commonly shift toward neutral or alkaline conditions, which can promote biofilm formation, increase protease activity, and delay healing [43]. Lactate is important because hypoxia and anaerobic metabolism are intensified in chronic or infected wounds, and elevated lactate has been linked with infected diabetic foot ulcers and delayed healing [43]. Uric acid is important because it is generated through purine metabolism under hypoxic and damaged-tissue conditions, and its concentration in the range of about 220–750 μM has been associated with wound severity, oxidative stress, and bacterial uricase activity [43]. Glucose is important because hyperglycemia, especially in diabetic wounds, supports bacterial proliferation, impairs angiogenesis, and disturbs normal healing dynamics, making glucose a clinically relevant metabolic target in multimodal wound platforms and bioelectronic sutures [50]. H_2_O_2_ and other ROS are important because moderate ROS participate in antimicrobial defense and wound signaling, whereas excessive ROS sustain inflammation, oxidative tissue damage, extracellular-matrix degradation, and chronicity [43]. Temperature is generally a physical rather than biochemical target, but it is frequently integrated with electrochemical biosensors because local temperature elevation may indicate inflammation or infection, while temperature also influences enzymatic kinetics, Nernstian pH response, diffusion, and wound metabolic activity [51].

### 3.3. Categorization of Electrochemical Biosensors

#### 3.3.1. Amperometric and Voltammetric Biosensors

Amperometric biosensors operate by applying a constant potential to the working electrode and measuring the resulting faradaic current generated by oxidation or reduction in the target analyte, enzymatic product, or redox mediator [43]. Chronoamperometry (CA) is a classical amperometric technique, whereas cyclic voltammetry (CV), differential pulse voltammetry (DPV), and square-wave voltammetry (SWV) are closely related current-based approaches in which the potential is scanned or pulsed to resolve peaks with improved selectivity and sensitivity [43]. In wound biosensing, amperometric and voltammetric formats are especially suitable for uric acid, lactate, glucose, oxygen, nitric oxide, H_2_O_2_, ROS, and pyocyanin because these biomarkers are either directly electroactive or can be coupled to enzyme-catalyzed redox reactions [43]. Pyocyanin (5-methyl-1-hydroxyphenazine) is a nitrogen-containing heterocyclic compound belonging to the phenazine class and is characteristically observed as a blue-green pigment produced by *Pseudomonas aeruginosa*. Its direct electrochemical detection is enabled by a reversible two-electron redox process at relatively negative potentials and an irreversible phenolic oxidation at more positive potentials, which produce characteristic potential-dependent voltammetric signals. Therefore, pyocyanin can be detected without requiring a separate biorecognition layer or extensive sample preparation [52]. Recent advances in amperometric and voltammetric wound biosensing have been characterized by the integration of selective biorecognition elements, low-potential redox mediators, nanostructured modified electrodes, flexible substrates, wireless readout systems, and wound-compatible sampling materials to improve analytical performance and enable wearable and in situ monitoring.

Kassal et al. developed a screen-printed smart bandage in which uricase was cross-linked with bovine serum albumin using glutaraldehyde, while a Prussian blue modified carbon electrode enabled amperometric detection of the enzymatically generated H_2_O_2_ at −0.3 V [53]. The sensor demonstrated a linear response over 100–800 μM uric acid, a sensitivity of −2.4 nA μM^−1^, and comparable calibration results using a laboratory electrochemical analyzer and a wearable potentiostat equipped with radio-frequency identification (RFID) and near-field communication (NFC) capabilities (RFID/NFC) enabled (R^2^ = 0.9967). Operation at a low negative potential limited interference from readily oxidizable compounds, while enzyme specificity further improved selectivity. The response remained between 95% and 102% of its initial value over 8 h, with an RSD of 2.02%, which was attributed to glutaraldehyde cross-linking, bovine serum albumin stabilization, and the protective chitosan layer. No significant response variation was observed after 80 bending cycles; however, the experiments were conducted in phosphate-buffered saline rather than authentic wound exudate, and stability beyond 8 h was not established [53].

Liu and Lillehoj subsequently fabricated embroidered electrodes by stitching carbon-coated polyester and Ag/AgCl-coated threads directly onto medical gauze to produce a mechanically deformable textile sensing platform [54]. The uricase-based sensor provided a linear amperometric response over 0–800 μM uric acid in simulated wound fluid (R^2^ = 0.995), with stable signals obtained approximately 1 min after sample application. Glucose, lactate, and creatinine produced negligible interference, and continuous measurements remained consistent for 7 h without an evident biofouling related signal loss. The sensor also retained its performance after 100 bending cycles and during measurement at a 90° bending angle. Nevertheless, its evaluation was limited to simulated wound fluid and short-term operation, and the authors suggested that an additional biocompatible coating, such as chitosan, would be required for longer monitoring periods [54]. Simoska et al. used flexible carbon ultramicroelectrode arrays and SWV to distinguish pyocyanin, uric acid, and nitric oxide according to their characteristic oxidation potentials in simulated wound medium [55]. These biomarkers provided complementary information on bacterial virulence, wound metabolism, and the host immune response. The respective limits of detection were 1.0 ± 0.5, 1.0 ± 0.4, and 0.20 ± 0.05 μM, with linear ranges of 1–250, 100–700, and 1–100 μM. SWV enabled discrimination from common electroactive interferents through peak potential separation, while the ultramicroelectrode configuration supported rapid mass transport and five-day electrochemical stability. The platform quantified pyocyanin released by *P. aeruginosa* and nitric oxide generated by stimulated macrophages in wound simulant. It also detected a decrease in pyocyanin from 25 ± 1 to 6.7 ± 0.3 μM within 30 min of Ag^+^ treatment, demonstrating its potential for monitoring antimicrobial response. However, broader and partially overlapping voltammetric peaks were observed in wound simulant, and the influence of wound pH and other matrix-dependent electroactive products remained a limitation [55].

Zhou et al. functionalized flexible laser-induced graphene (LIG) electrodes with molybdenum polysulfide (MoS_x_) to increase their electrochemically active surface area and improve phenazine detection by SWV [56]. In bacterial broth, the MoS_x_/LIG sensor achieved detection limits of 0.19 μM for pyocyanin and 1.2 μM for phenazine-1-carboxylic acid, corresponding to approximately 6.3- and 4.3-fold improvements, respectively, compared with platinum-modified LIG (Pt/LIG) electrodes. The detection limit for pyocyanin increased to approximately 1.3 μM when *Pseudomonas aeruginosa* was analyzed in wound-simulating medium, demonstrating the influence of sample complexity on analytical performance. A Nafion coating was applied to protect the electrode surface from biofouling and reduce direct interactions between the electrode material and bacterial cells, while *Escherichia coli* was employed as a phenazine-negative control. In agar-based biofilm models, phenazine production was detected approximately 12 h after inoculation at an initial bacterial density of about 5000 colony-forming units (CFU) mm^−2^; the detection time decreased to approximately 7 h when the initial bacterial density was increased tenfold. Although the two agar configurations simulated different spatial relationships between bacterial cells and the sensor, the platform was evaluated only using bacterial cultures and simulated wound models and therefore requires validation with clinical wound samples [56]. Thirabowonkitphithan et al. modified screen-printed carbon electrodes with a physically cross-linked multi-walled carbon nanotube/poly(vinyl alcohol) (MWCNT/PVA) hydrogel that enhanced electron transfer, absorbed wound fluid, and improved material biocompatibility [31]. The sensor showed a linear SWV response over 1–100 μM pyocyanin, with detection limits of 0.48 μM in phosphate buffer, 0.10 μM in lysogeny broth, 1.63 μM in simulated wound exudate, and 0.39 μM in Mueller–Hinton broth. Common wound interferents did not significantly affect pyocyanin detection, although sensitivity decreased in simulated wound exudate because of its higher matrix resistance. Selectivity was demonstrated using *E. coli* and *S. aureus* as negative controls, while pyocyanin released by *P. aeruginosa* was confirmed using mass spectrometry and quantified by high-performance liquid chromatography (HPLC). Direct detection was further demonstrated in controlled infected-burn models using ex vivo porcine skin and in vivo mice without sample pretreatment. These models represented an important advance toward wound applications; however, controlled bacterial inoculation and short experimental periods were employed, and performance in naturally infected human wounds was not established [31].

The main advantage of amperometric wound biosensors is that the magnitude of the current can be directly correlated with analyte concentration, enabling rapid quantitative readout in small volumes of wound exudate or simulated wound fluid [43]. Amperometric and voltammetric wound sensors have been implemented using a range of fabrication methods and electrode types, including screen-printed electrodes, embroidered textile electrodes, flexible carbon ultramicroelectrode arrays, laser-induced graphene electrodes, and hydrogel-modified electrodes [31,43,53,54,55,56]. These approaches can facilitate integration into wearable bandages and disposable wound patches, but they are not exclusive to current-based sensing; the choice of transduction mode depends on the target analyte, sensing chemistry, electrode configuration, and required readout. The principal limitations of amperometric and voltammetric wound biosensors include interference from coexisting electroactive compounds, electrode biofouling, enzyme instability, oxygen dependence, mechanical deformation, and matrix-dependent shifts in sensitivity and peak potential. Several strategies have been investigated to address these limitations. Enzymatic recognition and low-potential Prussian blue transduction have been used to reduce interference in uric acid sensors, while SWV has enabled electroactive biomarkers to be distinguished through their characteristic peak potentials [53,54,55]. Glutaraldehyde cross-linking, bovine serum albumin stabilization, and protective chitosan layers have improved enzyme retention and short-term operational stability [53], whereas embroidery, flexible ultramicroelectrode arrays, and laser-induced graphene have increased resistance to mechanical deformation [54,55,56]. Nafion coatings have been applied to limit biofouling and bacterial contact [56], and absorptive MWCNT/PVA hydrogels have facilitated direct sampling from wound surfaces [31]. Nevertheless, these measures have generally been validated for periods ranging from several hours to several days in buffer, simulated wound media, bacterial cultures, or controlled animal models. Protein-rich clinical exudate, variable pH and oxygen availability, polymicrobial communities, long-term enzyme degradation, and calibration drift therefore remain insufficiently addressed and require validation in naturally infected human wounds.

#### 3.3.2. Potentiometric Biosensors

Potentiometric biosensors measure the potential difference between a sensing electrode and a reference electrode under near-zero-current conditions, and the analytical signal is generated when ion activity, protonation state, or interfacial charge distribution changes at the electrode surface [48]. In wound monitoring, potentiometric transduction is most strongly associated with pH detection because hydrogen-ion activity changes during healing, inflammation, biofilm formation, and infection [48]. Polyaniline is one of the most widely used pH-sensitive materials because protonation and deprotonation of its nitrogen-containing backbone alter its redox state and interfacial potential across the wound-relevant pH range [48]. Potentiometric pH sensors are attractive for smart wound dressings because they require very low power, can operate without continuous faradaic current, and can be fabricated on flexible paper, textile, polymer, transparent Indium Tin Oxide (ITO), carbon, or suture-based platforms [48]. Their major advantages include low-cost readout, miniaturization potential, direct relation to wound alkalinization, and compatibility with wireless monitoring [48]. As shown in Figure 2A–D, a bandage-integrated potentiometric pH sensor was developed by Guinovart et al. through sequential screen-printing of a transparent ultraviolet-cured insulating layer, a silver/silver chloride (Ag/AgCl) conductive layer, a carbon working-electrode layer, and a skin-colored insulating layer [57]. In the assembled device shown in Figure 2E, polyaniline (PANi) was electropolymerized on the carbon working electrode as the pH-sensitive material, while a polyvinyl butyral (PVB)-based membrane was incorporated to form the all-solid-state reference electrode. As illustrated in Figure 2F, a near-Nernstian sensitivity of −57.9 ± 0.6 mV pH^−1^ was obtained using human-serum-based simulated wound samples over pH 7.93–4.07. Continuous monitoring of pH variations for 100 min was further demonstrated using a poly(ethylene glycol) (PEG) hydrogel to simulate the wound environment, as presented in Figure 2G. Low hysteresis, selectivity against physiologically relevant ions, and limited response variation under mechanical bending were also reported. Although the feasibility of incorporating potentiometric pH monitoring into a commercial bandage was demonstrated, the device was evaluated only under in vitro conditions, continuous monitoring was limited to 100 min, and wireless readout and validation in vivo/ex vivo wound models, and real patient wounds were not investigated [57].

A flexible and transparent potentiometric pH-monitoring platform was subsequently reported by Rahimi et al. [58]. PANi was electropolymerized on laser-patterned indium tin oxide (ITO) electrodes, and the disposable sensor was connected to a reusable, flexible, battery-free NFC module. A sensitivity of approximately −55 mV pH^−1^ was achieved over pH 4–10, with hysteresis below ±4 mV and signal drift equivalent to approximately 0.16–0.20 pH units over 24 h. Wireless measurements were obtained using a smartphone positioned within approximately 4 cm of the sensor. In an agarose-based microfluidic wound model inoculated with *Staphylococcus epidermidis*, an increase from approximately pH 6.8 to 8.6 was wirelessly recorded over 15 h, and the measurements were compared with those obtained using a commercial glass pH electrode. Thus, potentiometric wound monitoring was advanced from short-term wired measurements toward battery-free wireless detection of bacteria-associated alkalinization. However, only an in vitro proof of concept was demonstrated, and sufficient power could not be transferred to the NFC module when it was subjected to severe bending at a radius below 5 mm [58]. More recently, a pH-sensing suture was developed by Kim et al. through the integration of PANi-functionalized gold nanoparticle electrodes and PVB protected Ag/AgCl reference electrodes onto medical suturing thread [59]. This configuration was designed to enable pH monitoring in deeper wounds that cannot be adequately accessed using planar patches. A near-Nernstian sensitivity of 58.9 mV pH^−1^ was obtained over pH 4–10, together with negligible hysteresis, resistance to ionic and protein interference, and agreement with a commercial pH meter in complex biological media. Stable sensing performance was maintained during 10,000 bending cycles and after the suture was passed through porcine tissue, while sensitivity was preserved during 14-day stability testing. Following ex vivo evaluation in porcine tissue, wireless in vivo monitoring for up to 21 days was demonstrated in mouse incision and chronic-inflammation models. The recorded pH profiles were associated with the progression and resolution of inflammation and were supported by tumor necrosis factor-alpha (TNF-α) measurements. Nevertheless, bacterial infection was not directly investigated, and further improvement in mechanical compliance and biodegradability would be required to avoid a secondary procedure for removal of the sensing suture [59].

Overall, a clear progression has been demonstrated from short-term in vitro pH monitoring using bandage-integrated electrodes [57], through battery-free NFC-based monitoring in a simulated infected wound [58], to long-term wireless measurements in ex vivo tissue and living animal wound models using pH-sensing sutures [59]. However, validation in naturally infected human wounds has not yet been reported, and infection cannot be distinguished from inflammation or other healing related changes using pH alone. Therefore, long-term reference electrode stability, biofouling, temperature compensation, wireless reliability under mechanical deformation, integration with complementary biomarkers, and validation against established microbiological methods remain important requirements for clinical translation.

#### 3.3.3. Impedimetric Biosensors

In impedimetric biosensors, the frequency-dependent resistance and reactance of the electrode–electrolyte interface are measured in response to an applied alternating current perturbation. Information regarding charge transfer resistance, double-layer capacitance, diffusion, solution resistance, and interfacial film properties can be obtained from the resulting impedance spectrum [60]. Electrochemical impedance spectroscopy (EIS) has been widely applied to label-free wound monitoring because changes caused by bacterial attachment, biofilm development, biomarker binding, protein adsorption, and variations in wound-exudate composition can be detected without requiring an enzymatically generated electroactive product or an additional reporter label [49,61]. Consequently, repeated measurements can be performed using the same electrode, which is advantageous for continuous monitoring. Furthermore, interfacial and bulk processes can be examined across different frequency regions. However, biochemical specificity can be limited because biological binding, nonspecific adsorption, hydration, temperature, medium conductivity, and electrode geometry may produce overlapping impedance changes. Improved biochemical specificity has been achieved through the incorporation of selective biorecognition layers. Label-free impedance immunosensor was developed by Ciani et al. for Triggering Receptor Expressed on Myeloid Cells-1 (TREM-1), matrix metalloproteinase-9 (MMP-9), and N-(3-oxododecanoyl)-L-homoserine lactone (HSL), which represent inflammatory, tissue-degradation, and bacterial quorum-sensing processes, respectively [62]. Specific thiolated antibodies were immobilized on gold screen-printed electrodes, and surface blocking procedures were applied to reduce nonspecific protein adsorption. TREM-1 and MMP-9 were measured using direct immunoassays, whereas HSL was detected through a competitive assay because of its small molecular size. Detection was completed directly in protein containing mock wound fluid within 1 h without extensive sample preparation. Limits of detection of 3.3 pM for TREM-1, 1.1 nM for MMP-9, and 1.4 nM for HSL were achieved [62]. Through this biomarker combination, complementary information regarding host inflammation, extracellular-matrix degradation, and bacterial communication could potentially be obtained. Nevertheless, the biomarkers were evaluated using separate immunosensors in a defined mock wound matrix rather than an integrated array in clinical wound exudate, and the long-term stability and regeneration of the antibody-modified surfaces were not established.

As shown in Figure 3, a disposable screen-printed carbon-electrode impedance sensor on polyethylene terephthalate (PET) was developed by Hannah et al. for the real-time monitoring of wound pathogens in simulated wound fluid [61]. A mid-frequency decrease in normalized phase was produced by the growth of *Proteus mirabilis*, with a minimum near 600 Hz and a significant change detected after 2.5 h (Figure 3D). Similar phase changes were used to detect *Pseudomonas aeruginosa* after approximately 2 h (Figure 3E) and *Staphylococcus aureus* within 2–5 h (Figure 3F). An additional high-frequency phase peak was observed for *S. aureus*, indicating that time- and frequency-dependent features could potentially be used to differentiate bacterial responses. Baseline normalization was applied to reduce the effects of initial variation among disposable electrodes. However, partially similar mid-frequency responses were generated by the three microorganisms, and the experiments were conducted using separate monocultures in simulated wound fluid. Therefore, species level identification would require larger impedance response libraries and validated classification models incorporating multiple strains, bacterial loads, wound matrices, and microbial combinations. Such validation is particularly important because wound infections may be monomicrobial or polymicrobial, and the electrical response of one microorganism may dominate or obscure that of another [61,63,64]. The importance of this limitation was demonstrated in a polymicrobial model by Ward et al., in which a low-cost screen-printed carbon sensor was used to examine cultures containing *P. aeruginosa* and *S. aureus* in artificial sputum medium [63]. A characteristic reduction in low-frequency impedance and phase was detected after approximately 24 h when *P. aeruginosa* was grown either alone or in coculture. At 0.1 Hz, the impedance modulus was reduced from approximately 4.7 MΩ to 100 kΩ, and the response was attributed partly to pyocyanin and related electroactive phenazines. Baseline normalized measurements were used to facilitate comparisons among sensors and to distinguish cultures containing *P. aeruginosa* from sterile controls. However, the response produced by the coculture became similar to that of the *P. aeruginosa* monoculture because *P. aeruginosa* outcompeted *S. aureus*. By contrast, a significant impedance response was not produced by *S. aureus* alone under the investigated conditions. Therefore, although detection of a dominant electroactive organism was demonstrated, comprehensive identification of all organisms within a polymicrobial community was not achieved [63].

Time resolved analysis and validation using complementary measurements have been investigated to improve the interpretation of biofilm associated impedance changes. Dental-plaque-derived multispecies biofilms were monitored by Kumar et al. using poly(3,4-ethylenedioxythiophene)(styrene sulfonate) (PEDOT) modified electrodes, and the measurement frequency was optimized at 1 Hz [65]. Impedance changes of 2.5%, 35%, 50%, and 65% were correlated with biofilm volumes of 0.10 ± 0.01, 16.9 ± 2.2, 29.7 ± 2.3, and 38.6 ± 2.8 µm^3^ µm^−2^, respectively. A quantitative relationship between impedance and biofilm volume was established using confocal laser-scanning microscopy (CLSM), with a coefficient of determination of (R^2^ = 0.99). When the relationship was applied to an additional substrate, a prediction error of approximately 6% was obtained. Simultaneous pH measurements were also performed to relate biofilm accumulation to local metabolic acidification. Although a wound model was not used in this study, it was demonstrated that optimized single-frequency monitoring and independent microscopic validation could be used to convert a nonspecific impedance change into a quantitative estimate of biofilm development [65]. A related strategy was investigated by McGlennen et al., in which gold micro-interdigitated electrodes were modified with the conductive polymer poly(pyrrole)(styrene sulfonate) (PPy) and incorporated into a three-dimensional-printed flow cell [66]. Initial sensor variability was reduced by the PPy coating, and signal drift variability was restricted to approximately 2% over 18 h, compared with the substantially wider and bidirectional variation observed for unmodified electrodes. Through frequency analysis, 1.26 kHz was identified as an appropriate operating frequency for distinguishing biofilm-related responses from abiotic fluctuations. Under continuous flow at 1 µL min^−1^, a sigmoidal impedance decrease was produced by *P. aeruginosa* biofilm growth over 48 h, with the earliest significant difference from the abiotic baseline being observed after 12 h. Concurrent CLSM imaging was used to associate the impedance response with initial attachment, early proliferation, mature surface coverage, and biofilm detachment or regrowth. Nevertheless, the experiments were conducted under controlled culture conditions rather than in wound exudate, and the response became less distinguishable following extensive surface coverage because the effective sensing depth was limited by the electrode geometry [66]. Several strategies have been investigated to address the limitations of impedimetric biosensors, including selective biorecognition and surface blocking, baseline normalization, frequency optimization, conductive-polymer modification, and complementary imaging [61,62,63,64,65,66]. Nevertheless, biofouling, matrix-dependent signal variation, overlapping microbial responses, and limited clinical validation remain unresolved. Future studies should prioritize antifouling interfaces, multimodal sensing, and validation in polymicrobial and clinical wound samples. The sensing platforms, electrochemical techniques, target biomarkers, and analytical performance of representative electrochemical sensors and biosensors are compared in Table 1.

### 3.4. Electrochemical Sensing of Wound-Related Biochemical and Physiological Parameters

Electrochemical sensors and biosensors for wound infection monitoring are designed to convert specific biochemical or physiological changes within the wound microenvironment into electrical signals, thereby enabling quantitative and time resolved assessment of wound status at the point of care. The wound bed is characterized by dynamic alterations in metabolites, reactive oxygen species (ROS), pH, temperature, protease activity, and inflammatory mediators, all of which modulate bacterial proliferation, immune cell recruitment, extracellular matrix remodeling, and tissue regeneration. Traditional wound assessment, which relies on visual inspection and intermittent laboratory tests, provides only coarse, subjective, and temporally sparse information and therefore often fails to capture early biochemical signatures of infection or deterioration that arise between clinical visits [43]. By contrast, electrochemical sensing platforms embedded within dressings or bandages can continuously monitor key targets such as lactate, uric acid, glucose, hydrogen peroxide, and temperature directly in wound exudate, thereby supporting timely diagnosis of infection, precise tracking of healing trajectories, and data driven adjustment of therapy [43]. These targets are particularly compatible with electrochemical transduction because they are either inherently electroactive (for example, uric acid and hydrogen peroxide) or can be coupled to specific redox enzymes (for example, lactate oxidase and glucose oxidase), while temperature can be measured electronically via resistive elements that are readily co integrated with electrochemical electrodes in soft, wearable formats [67,68].

#### 3.4.1. pH Sensing

Wound pH has been established as a key physicochemical biomarker because the wound microenvironment undergoes a reproducible acid–base transition during normal healing, chronic inflammation, and bacterial infection [69]. Healthy skin and normally healing wounds exhibit a mildly acidic pH, whereas chronic and infected wounds usually shift toward neutral or alkaline values owing to exposure of interstitial fluid, bacterial metabolism, increased protease activity, and impaired oxygenation [69]. An increase in wound pH has been reported as an early indicator of infection, preceding obvious clinical signs, while alkaline conditions favor biofilm formation, enhance matrix metalloproteinase activity, reduce antimicrobial efficacy, and delay re-epithelialization [70]. In chronic lesions, pH values are commonly observed in the alkaline range of approximately 7.0–9.5, and severely infected wounds can reach values close to 10.0, in contrast to the progressive re-acidification seen in successfully healing wounds [71]. These findings have motivated the development of electrochemical pH biosensors that can provide real-time, quantitative, and dressing-integrated pH monitoring as an alternative to subjective visual inspection, traumatic dressing removal, and delayed microbiological assays. Compared with optical pH indicators, electrochemical pH sensors are particularly attractive for wound care because they offer fast response, high sensitivity, straightforward miniaturization, low-cost fabrication, and direct integration with wireless wearable platforms [48]. Several potentiometric electrochemical pH biosensors have employed polyaniline (PANI) as the proton-sensitive conducting polymer, since protonation and deprotonation of nitrogen sites in the PANI backbone produce reproducible open-circuit potential shifts with pH [57]. Guinovart et al. reported a bandage-based wearable potentiometric sensor in which electropolymerized PANI was deposited on a screen-printed carbon electrode embedded in a commercial adhesive bandage. In a poly(ethylene glycol) hydrogel wound model, the measured pH decreased from approximately 7.7 to 5.1 over 100 min following the addition of acidified human-serum-based wound simulant, with a relatively rapid decrease during the first 40 min followed by a slower approach to the final value [57]. Rahimi et al. subsequently developed a flexible PANI-based pH sensor array on polymer-coated paper for spatial wound pH assessment, where the sensor exhibited a linear response across pH 4–10 with an average sensitivity of about −50 mV pH^−1^ and response times on the order of tens of seconds, together with confirmed biocompatibility toward human keratinocyte cells [72]. In a later work, Rahimi et al. fabricated a flexible and transparent wound pH sensor by laser-patterning indium tin oxide and electropolymerizing PANI, which was connected to a near-field communication module to enable smartphone-based wireless readout; this sensor achieved a sensitivity of approximately −55 mV pH^−1^ over pH 4–10 and demonstrated stability suitable for simulated wound monitoring [58]. Zhang et al. further introduced a soft intelligent dressing that integrated PANI-based pH electrodes and temperature sensors on a stretchable elastomer substrate, providing a pH detection range of 4–10, response times below 6 s, and the ability to distinguish infected from non-infected wounds in a rat *Staphylococcus aureus* model based on local alkaline shifts [51].

Electrochemical pH monitoring has also been expanded toward textile, multimodal, and closed-loop smart dressings that better capture the complex wound microenvironment. Mariani et al. developed a reference-free, two-terminal pH sensor based on the semiconducting polymer poly(3,4-ethylenedioxythiophene) (PEDOT) doped with poly(styrene sulfonate) (PSS), denoted as PEDOT:PSS, and functionalized with iridium oxide particles (IrOx Ps) to continuously deliver wound exudate to the sensing region, and the system showed reversible pH response with a sensitivity of 59 ± 4 μA pH^−1^ over the clinically relevant pH range of 6–9 [73]. As illustrated in Figure 4A, a potential difference of −200 mV was applied between the two terminals, and the current flowing through the PEDOT:PSS/IrOx Ps film was measured as the analytical signal. The sensing mechanism was based on spontaneous electrochemical gating at the IrOx/PEDOT interface (Figure 4B). Under acidic conditions, Ir(IV) sites were reduced to Ir(III), accompanied by electron extraction from PEDOT and oxidation of PEDOT, which increased the hole concentration and current. The reverse process occurred under basic conditions, decreasing the PEDOT conductivity. Because the IrO_x_ particles acted as miniaturized potentiometric gates within the semiconducting film, a physically separated reference electrode was not required. As shown in Figure 4C, the sensor was integrated into a textile smart dressing containing absorbent gauze that functioned as both a fluid reservoir and a passive pump to transport simulated wound exudate to the sensing region. In the dynamic experimental setup shown in Figure 4D, an HPLC pump operating at 0.05 mL min^−1^ continuously delivered simulated wound exudate with different pH values across the sensor [68]. Mostafalu et al. developed a closed-loop smart bandage incorporating flexible pH and temperature sensors, a microcontroller, a heater, and thermo-responsive drug carriers, enabling wound status monitoring and electronically controlled, on-demand drug release for chronic wound management [74]. Sharifuzzaman et al. fabricated a stretchable multifunctional wound bandage based on an MXene-functionalized porous graphene scaffold, and the device allowed simultaneous monitoring of uric acid, pH, and temperature, with the uric-acid channel showing a linear range of 50–1200 μM and high sensitivity suitable for early infection assessment [75]. Xu et al. advanced the field by designing a fully integrated, battery-free, wireless smart dressing that simultaneously monitored pH, uric acid, and temperature via an NFC-powered electrochemical platform and coupled infection diagnosis with electrically controlled antibiotic delivery [76]. Kaewpradub et al. developed a fully printed bandage-based array combining a porous graphene/CNT pyocyanin sensor with a PANi/CNT pH sensor [77]. The simultaneous measurement of pyocyanin and pH provided an important analytical advantage because both the oxidation potential and current response of pyocyanin vary with pH. A partial least-squares model combining voltammetric and potentiometric data corrected this effect and achieved pyocyanin recoveries of 93.55–106.75% across simulated wound fluids of different pH values, whereas conventional single-variable calibration produced recoveries above 123% under acidic conditions and below 35% under alkaline conditions [77]. Potential interferents were evaluated in artificial wound fluid, and the pyocyanin sensor showed an RSD of 0.59% over 100 repeated measurements. Measurements of pyocyanin production by *P. aeruginosa* cultures were additionally supported by spectrophotometric measurements and viable-cell counting. Nevertheless, the platform was evaluated using artificial wound fluid and bacterial cultures rather than clinical wound exudate or in vivo wounds, so its performance under patient-specific biofouling and polymicrobial conditions remains to be established [77].

Overall, electrochemical pH biosensors are considered highly promising for wound infection monitoring because they can be integrated into flexible and textile dressings, provide rapid and quantitative electrical readouts, and interface seamlessly with wireless electronics, AI algorithms, and closed-loop therapeutic modules [48]. However, pH alone is not sufficiently specific for infection, as it is also influenced by wound stage, exudate composition, oxygenation and local inflammation, so pH sensing is best combined with complementary biomarkers such as temperature, uric acid, pyocyanin or lactate to increase diagnostic specificity [43]. Remaining technical challenges include long-term calibration drift, instability of miniaturized reference electrodes under mechanical deformation, biofouling by protein-rich exudate, temperature-dependent variation in the pH response, and possible biocompatibility issues with certain conducting polymers or nanomaterials. Consequently, future electrochemical pH biosensors for wound infection detection are expected to focus on robust solid-state reference systems, antifouling and biocompatible interfaces, integrated temperature compensation, multimodal analyte panels, and clinically validated pH thresholds that can reliably distinguish true infection from normal healing-related pH variations [48].

#### 3.4.2. Lactate Sensing

Lactate is the terminal product of anaerobic glycolysis and is generated in large amounts by hypoxic tissues and highly glycolytic immune cells such as activated neutrophils and macrophages, where it serves as both a metabolic substrate and a signaling molecule that influences angiogenesis, extracellular matrix production, and immune cell polarization within the wound bed. In acutely healing wounds, transient lactate accumulation promotes fibroblast proliferation, collagen synthesis, and neovascularization, whereas in chronic or infected wounds sustained high lactate concentrations often reflect persistent hypoxia, inadequate perfusion, and prolonged inflammatory cell activity, particularly in the context of diabetic ulcers. Clinical studies and recent sensor platforms indicate that wound exudate lactate typically lies in the low-millimolar range, commonly 0–4 millimolar in chronic wounds and up to 20–30 millimolar in severely ischemic ulcers, with elevated values being correlated with soft-tissue infection and impaired healing [68]. Because lactate is not electroactive at mild potentials, electrochemical detection usually relies on lactate oxidase (LOx), which catalyzes lactate oxidation to pyruvate and hydrogen peroxide; the hydrogen peroxide is then measured amperometrically using Prussian blue or noble-metal mediators, enabling sensitive, low-potential detection with good selectivity over common interferents in wound exudate [67,68]. Lactate is therefore a mechanistically and analytically attractive target for electrochemical wound sensors, as it reflects the combined effects of oxygenation, perfusion, and inflammatory activity while being amenable to enzyme-based amperometric transduction over clinically meaningful ranges. Reza et al. developed the multimodal CW-care patch for simultaneous monitoring of glucose, lactate, uric acid, Na^+^, K^+^, pH, and temperature [68]. This biomarker combination provided complementary information on wound metabolism, inflammation, electrolyte balance, and local physicochemical conditions, while the integrated pH and temperature sensors enabled algorithmic compensation of the metabolite and ion measurements. In artificial wound fluid, pH- and temperature-based correction changed the estimated glucose, lactate, uric acid, Na^+^, and K^+^ concentrations by approximately 22.5%, 16.0%, 11.6%, 26.5%, and 15.0%, respectively, demonstrating the importance of environmental compensation in multiplexed analysis [68]. A microfluidic collector with arrowhead patterns increased fluid-collection efficiency by approximately 6.5 fold and transported wound fluid to the sensors within 20 s. Matrix effects and biofouling were addressed using multilayer Nafion permselective membranes, while interference and cross-talk studies showed negligible responses to common nontarget species. The patch remained operational for approximately 750 min under dynamic artificial-wound-fluid conditions and was subsequently evaluated for multiday monitoring in a rat wound model. Glucose measurements were compared with a commercial glucose meter; however, equivalent reference-method validation was not reported for the other biomarkers, and clinical evaluation in human wounds remains necessary [68].

As shown in Figure 5A,B, Shirzaei Sani et al. developed a stretchable wireless bioelectronic patch integrating sensors for lactate, glucose, uric acid, ammonium, pH, and temperature with voltage-modulated electrodes for controlled drug delivery and electrical stimulation [67]. The lactate, glucose, and uric acid sensors were amperometric enzyme electrodes based on lactate oxidase, glucose oxidase, and uricase, respectively. As illustrated in Figure 5C, the enzymatic sensors comprised a nanostructured gold electrode, an electrodeposited Prussian blue layer, an enzyme/chitosan/CNT composite, and an outer polyurethane diffusion membrane. Prussian blue enabled low-potential detection of the enzymatically generated H_2_O_2_, whereas the polyurethane membrane controlled analyte diffusion, reduced matrix effects, and extended the linear response in wound fluid [67]. As shown in Figure 5D, the lactate sensor exhibited a sensitivity of 41.44 nA mM^−1^ and a linear response over 0–4 mM in simulated wound fluid. The sensor array maintained consistent responses under 15% uniaxial stretching and repeated mechanical deformation. In infected diabetic wounds, lactate and other infection-associated parameters increased after bacterial inoculation and decreased toward their pre-infection levels following treatment [67]. The electrical-stimulation component was independent of lactate detection and was employed therapeutically to promote tissue regeneration, while the voltage-modulated electrodes also controlled the release of the antimicrobial and anti-inflammatory peptide from the electroactive hydrogel [67]. Brown et al. developed an adhesive-free electronic extracellular matrix dressing integrating lactate, glucose, pH, oxygen, and temperature sensors within a stretchable and gas-permeable microfibrous scaffold [78]. This multimodal panel was intended to distinguish metabolic changes from variations in acidity, oxygenation, and temperature, thereby providing a broader assessment of wound status than an individual metabolite measurement. Importantly, the platform was evaluated not only in buffer but also in ex vivo exudate obtained from an infected stage-4 pressure injury. Glucose and lactate concentrations determined electrochemically were compared with a colorimetric assay, while the pH and oxygen sensors were calibrated against commercial probes. Testing in genuine wound exudate revealed clinically important matrix effects: lactate sensitivity decreased from −0.24 to −0.12 μA cm^−2^ mM^−1^, potential drift and diffusion limitations were observed, and protein deposition on the fibrous substrate confirmed biofouling. Although the porous scaffold facilitated fluid and gas transport, it did not eliminate surface fouling. Consequently, frequent in situ calibration and additional antifouling membranes were identified as necessary, and the single ex vivo specimen was insufficient to establish performance across heterogeneous patient wounds [78].

Textile-integrated lactate sensors have also been realized; for example, Liu and Lillehoj embroidered carbon-coated polyester yarns and Ag/AgCl threads onto medical gauze to form a three-electrode configuration, then immobilized lactate oxidase within a crosslinked hydrogel on the working electrode, achieving a linear amperometric response over approximately 0–10 millimolar lactate with detection limits below 0.1 millimolar in artificial wound exudate [79]. These embroidered sensors retained performance under bending and twisting and remained functional after integration into commercial multilayer dressings, illustrating a scalable and low-cost route to electrochemical lactate monitoring in clinical bandages. Simoska et al. fabricated flexible carbon ultramicroelectrode arrays (CUAs) on polyethylene terephthalate (PET), using square-wave voltammetry to simultaneously resolve the oxidation of pyocyanin, uric acid, and nitric oxide in wound-mimicking media and demonstrating detection limits in the low micromolar range, thereby establishing design principles for multianalyte ultramicroelectrode sensing that can be extended to lactate oxidase-based detection. Furthermore, wearable sweat lactate sensors based on laser-induced graphene or graphene oxide and lactate oxidase have reported sub-0.1 millimolar detection limits and fast response times, and their enzyme immobilization and antifouling strategies are now being adapted directly to wound exudate environments. Electrochemical lactate sensing provides a mechanistically grounded measure of hypoxia-related metabolic stress and inflammatory activity and can be implemented via mature lactate oxidase chemistries that deliver high sensitivity over the 0–10 millimolar range using well-established Prussian blue-mediated amperometric detection [67,68]. However, lactate oxidase exhibits strong temperature and pH dependence and requires adequate oxygen for reliable operation, so uncompensated environmental variations and ischemia can distort sensor outputs and complicate the attribution of lactate elevation specifically to infection rather than to non-infectious hypoxia. Chronic wound exudate is rich in proteins, cells, and proteases that promote electrode fouling, enzyme denaturation, and drift, while multiplex platforms must also avoid electrochemical cross-talk between lactate-derived hydrogen peroxide and other oxidase-based sensors sharing the same mediator or electrode [43,67]. Future research is likely to emphasize non-enzymatic or nanozyme-based lactate sensors, advanced antifouling coatings, and adaptive calibration schemes that fuse lactate, oxygen, pH, and temperature data, along with prospective clinical trials correlating continuous lactate measurements with microbiology, perfusion imaging, and healing outcomes across heterogeneous chronic wound populations.

#### 3.4.3. Uric Acid Sensing

Uric acid is the final product of purine metabolism in humans and is a major antioxidant in plasma and interstitial fluids; at the same time, elevated uric acid can promote inflammation and oxidative stress through crystal formation and activation of pattern recognition receptors, thereby exerting context dependent protective and deleterious effects. In wound exudate, uric acid typically spans approximately 220–750 micromolar in non-infected or healing wounds, whereas concentrations below about 200 micromolar have been associated with colonization by bacteria that express uricase and metabolize uric acid, while levels above 1 millimolar have been reported in chronic leg ulcers and reflect extensive tissue breakdown and oxidative stress. The specificity of this biomarker arises because humans lack endogenous uricase, while common wound pathogens such as *Staphylococcus aureus* and *Pseudomonas aeruginosa* produce uricase and can rapidly deplete uric acid, so shifts in exudate uric acid concentration carry information about bacterial colonization, infection severity, and necrotic burden. Electrochemical detection of uric acid is facilitated by its intrinsic electroactivity, allowing direct oxidation at carbon or metal electrodes, as well as by uricase based enzymatic schemes in which the resulting hydrogen peroxide is monitored amperometrically, with both approaches readily integrated into printed, textile, or flexible organic electrochemical transistor (OECT) architectures. Consequently, uric acid has emerged as a highly informative and analytically convenient target for electrochemical sensors and biosensors in wound infection monitoring [43]. As shown in Figure 6, Arcangeli et al. developed a fully textile OECT-based smart bandage for uric acid monitoring. The device was fabricated by screen-printing PEDOT:PSS on polyester–rayon gauze (Royal 100, Tenowo Italia S.r.l., Pieve Emanuele, Milan, Italy) to form the channel and gate in a cross-like geometry [80]. Upon gate polarization at +0.6 V, uric acid oxidation at the gate modulates the channel conductivity, enabling reference less detection via drain current changes. The sensor exhibited a limit of detection of 23–76 µM and a linear response from 100–1000 µM in simulated wound fluid, with minimal interference from common wound biomarkers. It maintained stable performance under mechanical bending, showed good device-to-device reproducibility, and demonstrated cytocompatibility [80]. Liu and colleagues developed embroidered electrochemical uric acid sensors by stitching carbon-coated polyester yarns as working and counter electrodes and silver-plated yarns as reference electrodes on medical gauze, followed by uricase immobilization in an ionically conductive hydrogel on the working electrode, resulting in a linear amperometric response over approximately 0–800 micromolar with detection limits near 100 micromolar in artificial wound exudate [79]. The embroidered sensors preserved performance under bending, twisting, and repeated wetting, and were compatible with multilayer dressings, confirming that textile manufacturing methods can yield robust uric acid sensors for clinical bandages [79]. Simoska et al. fabricated flexible carbon ultramicroelectrode arrays on PET via photolithography and pyrolysis and used square-wave voltammetry (SWV) to simultaneously resolve uric acid, pyocyanin, and nitric oxide oxidation peaks in wound-mimicking media, achieving uric acid detection limits around 5 micromolar and demonstrating that multianalyte electrochemical profiling of host and bacterial biomarkers is feasible on a single miniaturized platform [79].

Uric acid is advantageous as a sensing target because it is intrinsically electroactive, lies in a well-defined micromolar concentration window in wound exudate, and is mechanistically linked to both oxidative stress and bacterial metabolism, so changes in its level are clinically interpretable in terms of infection severity and necrosis. However, direct voltammetric detection of uric acid is subject to interference from ascorbic acid and other purine metabolites with similar oxidation potentials, and peak positions are sensitive to pH and electrode material, so selectivity must be carefully engineered via mediators, molecularly imprinted polymers, or enzymatic uricase layers [43]. Enzymatic uricase sensors can suffer from limited shelf life and operational stability under conditions of high ROS and protease activity, and the cost and complexity of enzyme stabilization may hinder widespread implementation if not addressed by low-cost immobilization strategies. Future developments will likely focus on integrating uric acid detection within multianalyte platforms that also monitor pH, temperature, and inflammatory cytokines, using OECT architectures and nanostructured carbon electrodes with antifouling coatings, and validating uric acid-based thresholds for infection and healing across different wound etiologies in multicenter clinical studies [43].

## 4. Raman Spectroscopy and SERS for Wound Infection Detection

Given the clinical need for rapid, non-invasive wound diagnostics and the limitations of current laboratory-based VOC detection methods, there is a growing need for point-of-care technologies that can identify infection signatures directly at the wound site [13]. One promising direction involves optical sensing methods capable of capturing bacterial VOC fingerprints with high chemical specificity and minimal sample preparation [81].

Among these optical approaches, SERS is emphasized in this review because it provides highly sensitive molecular fingerprinting that is complementary to electrochemical monitoring of wound-associated physicochemical and metabolic biomarkers [82]. Unlike optical approaches such as fluorescence or colorimetric sensing, which often rely on target-specific probes or changes in emitted or reflected intensity, SERS provides vibrational spectra containing chemically specific molecular information and can be implemented in a label-free format [83]. Its plasmon-mediated enhancement enables detection of low-abundance analytes while retaining the narrow spectral features of Raman spectroscopy, making SERS particularly attractive for multiplex analysis of bacterial cells, metabolites, virulence factors, and VOCs [84]. These characteristics, together with the development of portable Raman instrumentation and flexible plasmonic substrates, support its potential for point-of-care wound infection monitoring [85]. Nevertheless, SERS also presents important limitations, including signal variability caused by heterogeneous hotspot distributions and analyte–surface interactions, challenges in quantitative calibration and reproducibility, matrix interference in complex biological samples, and the need for standardized substrates and spectral databases [82]. Therefore, in this review, SERS is presented not as a replacement for other optical sensing approaches, but as a complementary molecular-sensing modality within multimodal wound diagnostic platforms.

### 4.1. Fundamentals of Raman Spectroscopy

SERS is a vibrational spectroscopy technique that achieves exceptionally high sensitivity by exploiting localized surface plasmon resonance (LSPR) in nanostructured metals such as gold and silver [86]. This plasmonic effect amplifies Raman scattering from molecules positioned near the metal surface, enabling the detection of extremely low VOC concentrations and providing chemically specific “fingerprints” that are advantageous for distinguishing bacterial species [87]. This technique also enables depth-resolved biochemical analysis; a recently developed snapshot depth-sensitive Raman system successfully differentiated wound edges from healthy rat skin, with classification accuracy improving as additional depth-resolved spectral data were incorporated [88]. Importantly, SERS platforms can be engineered into compact or wearable formats, require minimal sample preparation, and support real-time in situ measurements, for example when integrated with microneedles for minimally invasive sampling [89]. These features make SERS particularly well-suited for non-invasive wound infection detection and position it as a strong candidate for decentralized diagnostics where conventional tools such as GC-MS are impractical [90].

### 4.2. Raman vs. SERS: A Comparison

Raman spectroscopy and SERS both provide molecular vibrational information through inelastic light scattering but differ substantially in detection sensitivity. Conventional Raman spectroscopy is limited by its inherently weak scattering cross-section, resulting in low signal intensity relative to the much stronger elastic (Rayleigh) scattering [91]. SERS addresses this limitation by enhancing Raman scattering from molecules located at or near suitable nanostructured surfaces. Figure 7 illustrates the fundamental differences between Raman and SERS.

SERS enhancement is generally attributed to two principal mechanisms: electromagnetic and chemical enhancement. Electromagnetic enhancement arises primarily from LSPR in plasmonic nanostructures, commonly composed of Au or Ag [92]. Upon excitation, these structures generate intense localized electromagnetic fields, specifically at nanoscale junctions or “hotspots,” which enhance Raman scattering from molecules located within or near these regions [93,94]. Chemical enhancement arises from molecule–surface interactions, including chemisorption and charge-transfer processes, which can modify the Raman scattering response of adsorbed molecules [95]. Importantly, SERS does not uniformly enhance all Raman bands. The enhancement of individual vibrational modes depends on factors such as molecular orientation relative to the nanostructure surface and the direction of the local electromagnetic field. Consequently, surface selection rules may result in selective enhancement of particular vibrational modes and changes in their relative intensities compared with conventional Raman spectra [96,97].

### 4.3. SERS Analytes and Substrates for Wound Infection Monitoring

SERS has been applied to several analytes and parameters relevant to wound infection monitoring. These include wound pH, measured ratiometrically using pH-responsive Raman reporters [98,99], as well as bacteria associated with wound infection [100] and toxic bacterial metabolites such as pyocyanin produced by *P. aeruginosa* [101]. SERS has also been used to detect inflammatory cytokines such as interleukin-6 in diabetic wound exudate using a dual-mode electrochemical/SERS platform [102], while VOCs released through bacterial metabolism represent another relevant target (Section 4.4.3). These targets vary widely in molecular size, concentration, and sampling matrix, ranging from wound exudate to headspace gas. Consequently, substrate design is typically tailored to the target analyte. Recent reviews provide comprehensive discussions of SERS fundamentals [82] and plasmonic substrate design, including three-dimensional substrates and hotspot engineering [92], flexible Au- and Ag-based sensors [103], and metal–organic framework (MOF)-integrated substrates and their application to VOC analysis [104,105].

The performance of SERS in wound infection monitoring depends strongly on the plasmonic substrate, which governs both hotspot density and analyte accessibility; the substrates used can be broadly divided into metallic nanoparticle architectures and flexible or wearable platforms [103]. Anisotropic nanoparticles with sharp features, such as nanostars, nanotriangles and nanocubes, generate stronger local electromagnetic fields than spherical particles [106], and dense nanoparticle assemblies create interparticle gap hotspots; for example, a flexible PDMS substrate carrying a dense layer of Au nanoparticles gave an enhancement factor of 4.39 × 10^7^ [100]. Substrate homogeneity is equally important for reproducible measurements across sampling locations [107]. Porous shells such as MOFs and yolk–shell architectures, which concentrate analytes such as VOCs near the plasmonic surface, are discussed in Section 4.4.3.

### 4.4. SERS-Based Detection Strategies for Wound Infection

SERS-based approaches for wound infection detection can be broadly categorized according to the target of detection: pH sensing, direct detection of bacteria and bacterial metabolites, and VOC-based detection.

#### 4.4.1. SERS-Based pH Sensing

Wound pH is a key indicator of the wound microenvironment: healthy skin is mildly acidic, whereas chronic and infected wounds frequently shift to neutral or alkaline values that favor bacterial proliferation [98,99]. SERS-based pH sensing can rely on pH-responsive Raman reporters such as 4-mercaptobenzoic acid (4-MBA), whose carboxyl group undergoes reversible protonation and deprotonation as pH changes [98,99]. This alters the relative intensities of characteristic Raman bands, enabling ratiometric pH measurement, for example by using a pH-insensitive band as an internal standard to reduce dependence on absolute SERS intensity [99].

Zhu et al. incorporated 4-MBA-functionalized yolk–shell Au nanostar@ZIF-8 nanocomposites into hydrogel microneedles. The ratio of the SERS bands at 1134 and 1178 cm^−1^ showed a linear response over a pH range of 4–8 in artificial wound exudate (R^2^ = 0.97), while the yolk–shell design increased SERS intensity by 217% compared with core–shell analogues [98]. Layered filtration by the microneedle structure and molecular sieving by the ZIF-8 shell helped protect the plasmonic surface from biofouling, enabling in situ SERS pH mapping in infected mouse wounds during treatment [98]. Pan et al. incorporated 4-MBA-modified silver nanorods, with a plasmon resonance matched to 1064 nm excitation, into silk fibroin/hyaluronic acid microneedles. The pH-dependent SERS signal showed a linear response over a pH range of 4.2–9.2 (R^2^ = 0.97) and remained linear in wound exudate [99]. In MRSA-infected mouse wounds, SERS-derived pH measurements agreed with those obtained using a conventional pH meter, while a random-forest model trained on daily pH features classified infection severity into four grades with an overall accuracy of 94.64% [99].

Both platforms also integrate sensing and therapy within the same device. In the system developed by Zhu et al., the yolk–shell nanocomposites provide photothermal heating and Zn^2+^-mediated reactive oxygen species generation, which inhibited *P. aeruginosa* and *S. aureus* and promoted wound healing in mice [98]. In the Pan et al. system, the silver nanorods act as photothermal agents under second near-infrared (NIR-II) irradiation, while machine-learning analysis of the SERS pH data guides photothermal treatment, creating a closed diagnostic–therapeutic loop [99]. A similar closed-loop strategy has been demonstrated using a non-SERS optical readout, in which self-sterilizing MOF-hydrogel microneedles were combined with pH-sensitive fluorescent reagents, smartphone imaging, and machine-learning analysis [108]. These theranostic microneedle platforms have primarily been validated in buffer, artificial wound exudate, and animal models [98,99,108]. Further investigation is therefore needed to establish long-term reporter stability, calibration reliability under biofouling conditions, and performance in human wounds.

#### 4.4.2. Direct Detection of Bacteria and Bacterial Metabolites

SERS-based bacterial detection can follow either label-free or labeled strategies. Label-free approaches measure the intrinsic SERS spectrum of bacterial cells and their surrounding molecular environment, providing characteristic chemical fingerprints without target-specific labels [84]. In contrast, labeled approaches use SERS nanotags containing Raman reporters, often combined with recognition elements such as antibodies or aptamers, to generate target-specific reporter signals [84]. Recent reviews provide detailed discussions of SERS substrates, labeling strategies, and assay formats for bacterial detection [84,85]. The following discussion considers these approaches in the context of wound infection monitoring.

For example, antimicrobial and plasmonic Ag–Cu_x_O nanostructures enabled label-free identification of five bacterial species, including *E. coli* and *S. aureus*, at 10^3^ CFU/mL [109]. Bacterial disintegration at the nanostructured surface released intracellular components that contributed to the SERS signal [109]. Label-free SERS spectra of different bacterial species can exhibit distinct peak positions and relative intensities, enabling species discrimination using conventional spectral analysis and machine-learning approaches. Machine-learning analysis of bacterial SERS spectra, including species identification and discrimination of antibiotic-resistant strains, is discussed in Section 5.2. Previous studies have shown that many dominant peaks in bacterial SERS spectra originate from purine metabolites released during bacterial metabolism, while the overall combination of spectral features remains species-dependent [110]. For example, Premasiri et al. reported a prominent SERS band at approximately 737 cm^−1^ in *S. aureus*, closely corresponding to the 739 cm^−1^ ring-breathing mode of adenine, supporting the contribution of extracellular purine metabolites to bacterial SERS fingerprints [110].

In contrast to these label-free approaches, labeled SERS strategies use recognition elements and Raman reporters to provide target-specific detection in complex wound samples. For wound monitoring, flexible plasmonic substrates can facilitate direct sampling from irregular wound surfaces. Zhou et al. developed a flexible PDMS membrane functionalized with a dense layer of ~40 nm AuNPs and bacteria-specific antibodies, which served as both a bacterial-capture surface and a plasmonic component of the SERS platform [100]. When combined with Au@Ag core–shell SERS probes carrying Raman reporter molecules in a sandwich immunoassay format, the platform achieved LODs of 2.79, 3.42, and 4.52 CFU/mL for *E. coli*, *S. aureus*, and *P. aeruginosa*, respectively, and was evaluated in infected mouse wounds and clinical wound samples [100].

Labeled probes can also use reporters with Raman bands in the silent region (approximately 1800–2800 cm^−1^), where endogenous biomolecules produce minimal interference, enabling low-background detection in wound tissue; such probes have also been integrated with therapeutic functions. Deng et al. developed Au nanoparticles coated with Prussian blue, which acts both as a silent-region Raman reporter (2133 cm^−1^) and as a near-infrared photosensitizer, and loaded them with the antibiotic colistin sulfate (Figure 8) [111]. The probe quantified *E. coli* and *P. aeruginosa* over 10^3^–10^7^ CFU/mL, with LODs of 127 and 143 CFU/mL, respectively, while the combined photodynamic and antibiotic treatment reduced Gram-negative bacteria by more than 90% at a reduced antibiotic dose [111]. In normal and diabetic mouse wound models, SERS imaging tracked bacterial load during treatment. In 36 clinical wound samples (21 Gram-positive and 15 Gram-negative infections), the SERS signal distinguished Gram-negative from Gram-positive infections with an area under the ROC curve of 0.94 [111].

Guo et al. used aptamer-functionalized silver@copper-containing Prussian blue core–satellite nanostructures, whose cyano band at 2086 cm^−1^ enabled near-zero-background SERS imaging [112]. The *S. aureus*-targeting aptamer provided an LOD of 10 CFU/mL for *S. aureus*, compared with 1 × 10^5^ CFU/mL for *E. coli*. SERS imaging of residual bacteria was then used to guide near-infrared-triggered release of silver, copper, and iron ions for the treatment of infected diabetic wounds and keratitis in mice [112]. Together with the flexible capture platform developed by Zhou et al. [100], these studies demonstrate a progression from spectral identification of cultured bacteria toward in situ monitoring of wound infection, although validation has so far been limited to animal models and relatively small sets of clinical samples.

Beyond direct bacterial identification, SERS can also detect bacterial metabolites such as pyocyanin (PYO), a phenazine metabolite produced by *Pseudomonas aeruginosa*. Tanaka et al. developed a portable SERS sensor for PYO detection in simulated wound fluid using Ag, AgAu, and Au nanoparticles, with AgNPs providing the highest sensitivity (LOD = 1.1 μM), compared with 10.9 μM for AgAuNPs and 17.7 μM for AuNPs [101]. The characteristic PYO band at 1358 cm^−1^, attributed to C=C and C–N stretching and CH_3_ bending, was used for quantitative analysis in simulated wound fluid, while a band at 1636 cm^−1^, shifted from the 1608 cm^−1^ PYO signature, was used for analysis of cultured *P. aeruginosa* supernatants because of matrix interference near the 1358 cm^−1^ region [101]. The AgNP-based approach was further adapted to cotton-swab sampling, enabling SERS measurement within 5 min, and PYO detection was validated using cultured *P. aeruginosa* [101].

#### 4.4.3. SERS-Based VOC Detection: A Promising Direction for Wound Monitoring

VOC-based SERS detection is attractive for wound monitoring because volatiles diffuse from the wound into the surrounding headspace, allowing non-contact sampling without fluid collection (Figure 9) [13,113]. Evaluating its feasibility, however, requires comparing the VOCs actually reported in infected wounds with the analytical performance of existing SERS platforms.

**Wound VOC evidence.** A recent systematic review identified 11 studies of VOCs in human wounds, describing 105 distinct compounds; only dimethyl disulfide (DMDS) was reported in three studies, and the review noted substantial methodological heterogeneity [114]. DMDS was among the compounds discriminating chronic leg ulcers from healthy skin [115]. It was also detected in 14 of 17 infected surgical-site tissue samples and in none of 13 uninfected samples [116], and appeared only in severely infected wounds in a clinical swab study [117]. The same tissue study detected acetophenone in four infected samples, three of which contained *P. aeruginosa* [116], while hydrogen cyanide (HCN) was associated with *P. aeruginosa* biofilms in an ex vivo human wound model [118]. In a pilot study of 26 clinical wound swabs, short- and branched-chain fatty acids were identified as the strongest discriminators of infected from non-infected wounds [117].

Because the wound studies considered here report compound occurrence or relative abundance rather than absolute headspace concentrations, quantitative context was instead drawn from bacterial culture headspace, where, for example, DMDS reached ~10–14 ppbv above *S. aureus* and *P. aeruginosa* cultures and 2-butanone reached ~41 ppbv (maximum median) above *P. aeruginosa* [119]. A collagen wound biofilm model analyzed by SIFT-MS, which provides absolute concentrations, offers a closer approximation to the wound environment: HCN and dimethyl sulfide discriminated *P. aeruginosa* biofilms, and 2-methylbutanal reached 40–110 ppb above biofilms of two *S. aureus* strains and exceeded 1000 ppb for a third [120].

**SERS detection of bacterial headspace VOCs.** SERS detection of bacterial volatiles has been demonstrated in culture headspace. Ag and Au nanoparticle films detected DMDS, via its surface-bound methyl sulfide product, above cultures of six bacterial species, with an additional cyanide band observed for *P. aeruginosa*; notably, no bands from other headspace metabolites were reported, reflecting the preferential binding of sulfur- and cyanide-containing species to the metal surface [121]. Time-dependent DMDS signals have also been quantified above bacteria growing on agar [122], and HCN was detected in the headspace of *P. aeruginosa* isolates from cystic fibrosis patients [123]. However, these studies were performed on cultures rather than wounds, and gas-phase detection limits at biologically relevant levels were not established: DMDS was calibrated in liquid media [121], and gaseous HCN was tested only at 5 ppm, with ppb-level estimates derived from aqueous calibration [124].

**Comparison and outlook.** Comparison with gas-phase SERS platforms suggests that analytical sensitivity is strongly compound-dependent (Table 2). Several aldehyde- and aromatic-compound sensors summarized by Meng et al. achieve low-ppb detection [87], and acetophenone has been detected at 1 ppb using a TiO_2_ nanotube/Au@ZIF-8 substrate [125]. In contrast, a MIL-100(Fe)-coated Au nanostar sensor reported an LOD of 4.87 ppm for 2-butanone [126], approximately two orders of magnitude above the level measured in *P. aeruginosa* culture headspace, and no gas-phase SERS data were identified for short- or branched-chain fatty acids. Because the wound studies evaluated here do not provide absolute headspace concentrations, culture and wound-model values offer only approximate context and cannot define clinical sensitivity requirements. Direct SERS analysis of VOCs emitted from infected wounds therefore remains comparatively underexplored. Progress will require absolute quantification of wound-headspace VOCs together with SERS substrates that improve analyte capture, selective enrichment in complex mixtures, and signal reproducibility [87], needs that motivate the pre-concentration strategies discussed below.

**MOF–SERS substrates for VOC capture.** A key challenge in SERS-based VOC detection is the limited adsorption of trace gaseous analytes on plasmonic surfaces. MOFs, which possess high surface areas and tunable pore structures, can be integrated with plasmonic nanoparticles to concentrate VOC molecules near SERS-active regions and improve detection sensitivity [104]. MOF–SERS architectures may also provide molecular selectivity through tunable pore size and surface chemistry and can improve nanoparticle stability [104,105].

A recent review summarizes how MOFs contribute to SERS-based analysis (Figure 10B) [105]. First, their porosity accumulates analyte molecules close enough to the metal surface to be probed by the confined plasmonic field. Second, the framework can act as a molecular sieve, providing selectivity based on pore size and surface chemistry [105]. Its local chemical environment can also reduce competition from water for adsorption sites; hydrophobic ZIF-8 micropores are particularly useful in this regard, and ZIF-8-based SERS sensing of breath aldehydes has been reported to be unaffected by humidity [104,105]. Third, the chemical, thermal and mechanical stability of MOFs helps protect the metal surface from oxidation, poisoning and non-specific interactions. In addition, the higher refractive index of the MOF can extend the decay length of the plasmonic field, and matching the MOF band structure to that of the analyte can add a charge-transfer contribution to the enhancement [105]. These features correspond directly to the limitations identified above for wound-relevant VOCs: weak adsorption of small molecules that lack thiol or cyanide groups, competition from abundant background volatiles, and the high humidity of wound headspace.

Several MOF–SERS platforms illustrate these capabilities. Xie et al. developed AuNPs@MIL-101(Cr) composites for VOC detection, reporting detection limits of 5–75 ppm (Figure 10A) [127], while Au nanostars coated with MIL-100(Fe) detected gaseous 2-butanone, eucalyptol and isopropanol, salivary indicators of gastric cancer, with LODs of 3.07–8.26 ppm [126]. Au nanocube@ZIF-8 core–shell nanoparticles with a 10 nm shell detected benzaldehyde through 4-aminothiophenol capture with a reported LOD of 2.5 ppb [128]. However, factors such as MOF shell thickness and porosity can affect SERS performance by influencing VOC diffusion to the plasmonic surface and the resulting electromagnetic enhancement [105,129]. These considerations are especially relevant when adapting MOF–SERS substrates for complex and humid wound environments.

**Yolk–shell architectures.** These nanostructures provide an additional strategy for improving VOC capture: a metallic nanoparticle is separated from a porous outer shell by an internal void that can act as a gas-trapping reservoir, allowing VOCs to accumulate near the plasmonic surface while reducing the diffusion constraints associated with compact core–shell structures [130]. The porous shell may also improve substrate stability and limit competition from water vapor [104,105], which could be advantageous in humid wound environments, although this has not yet been evaluated in wound headspace.

Several studies have demonstrated the potential of yolk–shell and related MOF-based SERS substrates for VOC detection. Shi and Fang developed MesoAu@ZIF-8 nanostructures that combined a mesoporous gold core with a ZIF-8 shell, achieving a detection limit of 0.32 ppb for gaseous benzaldehyde [131]. Other architectures, including Au nanostar@MOF composites and TiO_2_NTs/Au@MOF nanocomposites, have demonstrated improvements in molecular sieving, substrate stability, and signal reproducibility [125,132]. These studies illustrate how porous plasmonic architectures can improve VOC capture and SERS detection, although their translation to wound monitoring requires evaluation in wound-relevant matrices and clinical settings. Table 3 summarizes the reported performance of representative MOF- and yolk–shell-based SERS substrates for VOC detection.

### 4.5. Practical Considerations for Wound Applications

Despite these advancements, SERS-based wound sensing systems face several challenges, including signal variability, substrate stability, and integration into clinically practical platforms. Flexible and wearable SERS substrates that conform to irregular wound surfaces, breathable materials that improve patient comfort, and robust power and data transmission solutions are all important for real-world implementation. The high sensitivity and molecular specificity of SERS, however, make them promising tools for early and non-invasive infection detection.

## 5. Artificial Intelligence and Data Analytics

The integration of electrochemical biosensors, SERS platforms, and wearable wound dressings generates complex, high-dimensional datasets that reflect simultaneous changes in host physiology and microbial activity. These datasets include time-resolved electrochemical signals (pH, lactate, uric acid, glucose), spectral fingerprints from SERS-based VOC detection, and environmental parameters such as temperature and humidity. Translating these multimodal signals into clinically actionable information requires advanced data analytics and artificial intelligence (AI) techniques capable of pattern recognition, feature extraction, and predictive modeling. In wound infection monitoring, AI does not merely process data but functions as an interpretive layer that links raw biosensor outputs to infection states, pathogen identity, and healing trajectories.

### 5.1. Machine Learning in Electrochemical Sensing

In electrochemical wound biosensing platforms, chemometric and machine-learning approaches are used to analyze complex, multi-biomarker temporal signals for infection classification and wound-status assessment. Chemometric methods such as principal component analysis (PCA) are commonly applied to combined electrochemical outputs to reduce data dimensionality, identify patterns and correlations among biomarkers, and visualize the separation between different wound states. In contrast, supervised machine-learning methods can use extracted features or temporal signal profiles to classify infection status or predict wound outcomes.

Supervised learning has begun to be applied to wearable wound sensors. For example, a deep artificial neural network trained on the pH-responsive voltage output of a flexible sensor integrated with a wound dressing was used to monitor chronic skin wounds and recognize their healing stage [133]. Classifiers such as support vector machines and random forests can integrate multiple sensor inputs for classification, while random-forest models can additionally provide estimates of feature importance. In a multimodal patch, they could therefore identify which combination of biomarkers (e.g., elevated pH, rising lactate and increasing temperature) best predicts infection onset, and give greater weight to the more informative sensor channels during real-time monitoring. Such multi-biomarker electrochemical classifiers remain at an early stage, however, and have not yet been validated across patients.

### 5.2. Machine Learning in SERS Sensing

SERS-based wound diagnostics generate complex spectral fingerprints representing VOCs, bacterial metabolites, and biochemical byproducts from infected tissue. These spectra contain overlapping peaks, baseline drift, and patient-specific variability, making manual interpretation impractical in real-world settings [134].

Deep learning models are therefore increasingly used to map raw or minimally processed SERS spectra directly to bacterial identity without explicit peak assignment, and to distinguish antibiotic-resistant from susceptible strains [134], as illustrated by the examples below. Autoencoders can additionally compress spectra into compact feature representations before classification [135]; in wearable wound systems, such preprocessing could help compensate for signal variability caused by moisture, substrate heterogeneity and environmental fluctuations, although this has not yet been demonstrated. Beyond spectroscopy, CNNs are already established in wound monitoring: they have been used to classify wound images for peripheral arterial disease and healing potential [136], and to interpret colorimetric wound sensors, evaluating healing and infection status from the color of a pH-responsive hydrogel dressing with 94.47% accuracy [137] and distinguishing normal from perturbed wounds with 96.3% accuracy using a five-biomarker sensor patch [138]. Their direct application to SERS spectra acquired from wounds has been more limited; the wound-specific SERS example identified here used a random-forest model on SERS-derived pH readings from infected mouse wounds to grade infection severity (Section 4.4.1) [99].

For bacterial identification, integrating SERS with machine learning has substantially improved classification accuracy and enabled automated analysis of complex spectral datasets. For example, SERS combined with deep learning achieved classification accuracies of up to 98% for distinguishing *E. coli* from *S. epidermidis* in complex media without any bacterial separation steps [139]. Moreover, a CNN trained on 17,149 SERS spectra from 30 bacterial species achieved high prediction accuracy for bacterial identification at both the genus and species levels [140]. In addition to species identification, SERS can also differentiate antibiotic-resistant strains. For instance, a stacked autoencoder-based deep neural network classified methicillin-resistant *S. aureus* (MRSA) from methicillin-sensitive *S. aureus* (MSSA) with 97.66% accuracy, as demonstrated by the ROC curve and confusion matrix in Figure 11 [135].

### 5.3. Multimodal Data Fusion and Predictive Modeling

A key limitation of single-sensor wound monitoring systems is that no individual biomarker reliably distinguishes between inflammation, colonization and infection. Multimodal data fusion addresses this limitation by integrating heterogeneous signals into a unified diagnostic framework. Multi-biomarker fusion has already been demonstrated with colorimetric sensing: a neural network combining five wound biomarkers (temperature, pH, trimethylamine, uric acid and moisture) classified healing versus non-healing wounds in rats with up to 97% accuracy [138].

In a patch combining electrochemical and SERS sensing, electrochemical sensors would provide continuous time-series data for host-response markers such as pH, lactate, uric acid, glucose and temperature, while SERS would add intermittent but highly specific bacterial and VOC information; fusion algorithms would align these data temporally and learn their joint relationship. For example, a simultaneous rise in wound pH and lactate may indicate metabolic stress, but combined with SERS signatures characteristic of *P. aeruginosa* it would point more strongly to active infection, whereas stable host markers without pathogen-specific signals would favor normal healing. Such fusion could potentially improve diagnostic specificity and support earlier detection of infection by combining complementary host- and pathogen-derived information. Electrochemical–SERS fusion has so far been demonstrated for a single biomarker, with a self-attention neural network combining both modes to quantify IL-6 in diabetic wound exudate (Section 5.4) [102], whereas multi-biomarker electrochemical–SERS classification of wound infection status was not identified among the studies evaluated in this review.

Digital twins could extend multimodal wound monitoring from static diagnosis to dynamic prediction. In this framework, each patient’s wound is represented by a continuously updated virtual model into which sensor data are assimilated, allowing AI models to forecast future wound states, such as risk of infection, healing delay or response to treatment, under different therapeutic scenarios. A digital-twin framework for chronic wound management has so far been demonstrated using wound images, with a generative model forecasting changes in wound tissue composition [141]; incorporating continuous electrochemical and intermittent SERS data streams would be a natural next step. A rising lactate trend combined with persistently alkaline pH and emerging VOC signatures could then trigger an early warning of infection before visible symptoms appear, supporting a shift from reactive treatment to proactive, personalized wound management.

### 5.4. Multimodal Biosensing Platforms for Wound Infection Detection

Wound infection is a complex biological process involving simultaneous changes in host metabolism, immune response, and microbial activity. Because individual biomarkers may also vary with non-infectious processes, reliance on a single biomarker can limit diagnostic specificity. For instance, elevated pH alone cannot distinguish between infection and normal inflammatory healing phases. Similarly, increased lactate may reflect hypoxia rather than bacterial infection, and VOC signatures alone may not capture host inflammatory status. These overlapping effects limit the diagnostic reliability of single-signal systems. Multimodal sensing overcomes these limitations by integrating complementary biomarkers that reflect both host response and pathogen behavior. This combined perspective enables more accurate differentiation between sterile inflammation, colonization, and active infection.

Recent advances in wearable biosensing have enabled integration of multiple sensing modalities into single wound dressings. These systems typically combine electrochemical sensors for continuous biochemical monitoring with auxiliary physical or optical sensors for environmental or metabolic assessment.

Common multimodal configurations include pH and temperature sensors for inflammation tracking, pH and uric acid sensors for metabolic assessment, and lactate and glucose sensors for diabetic wound monitoring. Representative wearable platforms evaluated in infected animal wound models, as discussed in Section 3.4, have demonstrated simultaneous monitoring of multiple biochemical and physiological wound parameters during infection and treatment [67,68]. More advanced systems combine electrochemical sensing with optical readouts, enabling complementary chemical and molecular information acquisition [142]. For example, Chen et al. developed a dual-mode microfluidic platform combining electrochemical sensing and SERS for IL-6 detection in diabetic wound exudate. The electrochemical and SERS modes achieved detection limits of 0.085 and 0.047 pg/mL, respectively. A neural network incorporating a self-attention mechanism was used to integrate information from both sensing modes, achieving quantitative accuracy of up to 99.8% over an IL-6 concentration range of 0.05–1000 pg/mL [102]. Although most existing platforms remain in the research or preclinical stage, they demonstrate the feasibility of integrating multiple sensing channels into flexible and biocompatible wound dressings.

Electrochemical biosensors continuously track host-derived biochemical changes such as pH, lactate, uric acid, and glucose in wound exudate, providing real-time insight into metabolic state, inflammation, and tissue stress. In contrast, SERS provides molecular fingerprints of bacteria and their metabolites, supporting pathogen identification and, potentially, resistance profiling [100,135], whereas VOC-based SERS detection for wounds remains at an early stage (Section 4.4.3). Integrating these two modalities allows simultaneous monitoring of host response and microbial behavior within the same wound environment. Such a dual-information framework could improve diagnostic confidence by combining host-response and microbial information, particularly when individual biomarkers show ambiguous changes.

## 6. Conclusions, Challenges and Future Work

Future wound diagnostic systems are expected to combine electrochemical biosensors, SERS, and artificial intelligence within a single wearable platform. Electrochemical sensors provide continuous monitoring of host-derived biomarkers, such as pH, lactate, glucose, uric acid, reactive oxygen species, and temperature. Simultaneously, SERS enables highly specific detection of bacterial cells, virulence factors, and VOCs through their molecular fingerprints. By integrating these complementary sensing modalities and their associated data, multimodal platforms can provide a more comprehensive picture of the wound microenvironment than either technique alone. Artificial intelligence can then analyze these datasets to identify complex patterns associated with infection, supporting earlier diagnosis, personalized treatment, and improved clinical decision-making.

Electrochemical sensing and SERS offer complementary advantages and limitations for wound monitoring. Electrochemical biosensors are well suited to continuous, quantitative, and low-power monitoring of soluble wound biomarkers and can be readily integrated into flexible and wearable platforms; however, their performance can be affected by biofouling, sensor drift, and interference in complex wound exudate [40]. In contrast, SERS provides highly sensitive and chemically specific molecular fingerprints that enable detection of bacterial cells, metabolites, and VOCs, but its performance depends strongly on substrate reproducibility, analyte–surface interactions, and spectral variability [84,97]. Nanomaterials play an important role in both modalities by increasing electroactive surface area and facilitating charge transfer in electrochemical sensors, while plasmonic Au and Ag nanostructures generate localized electromagnetic enhancement and molecularly specific SERS signals [75,92]. Flexible nanostructured substrates further support conformal sampling from irregular wound surfaces, facilitating integration with wearable diagnostic formats [100].

Advances in miniaturized potentiostats, portable Raman spectrometers, and wearable electronics are also important for translating these sensing approaches from laboratory settings toward point-of-care wound assessment [101]. Portable instrumentation can enable measurements closer to the patient, while AI provides an additional analytical layer for processing multivariate electrochemical signals and complex SERS spectra, extracting diagnostically relevant features, and integrating complementary sensing outputs [101,102,143]. Therefore, continued advances in these areas may facilitate clinical translation by improving sensing performance, enabling point-of-care operation, and supporting the interpretation of complex multimodal datasets.

Such integrated systems have the potential to shift wound management from reactive treatment toward proactive and personalized care. Continuous monitoring could enable earlier identification of infection before infection becomes clinically apparent, allowing clinicians to intervene sooner and potentially preventing progression to severe infection, hospitalization, or limb amputation. AI-assisted decision support could also reduce unnecessary antibiotic prescribing by distinguishing infection from sterile inflammation, thus supporting antimicrobial stewardship. Furthermore, wearable multimodal platforms capable of wireless data transmission may facilitate remote patient monitoring, reducing clinic visits while improving access to specialized wound care for patients in home-care and rural settings. Although important challenges remain before widespread clinical implementation, the convergence of electrochemical biosensing, SERS, wearable electronics, and artificial intelligence represents a promising pathway toward next-generation point-of-care wound diagnostics. By integrating complementary sensing modalities with intelligent data analytics, multimodal platforms have the potential to enable earlier infection detection, continuous monitoring, personalized treatment strategies, and improved patient outcomes while reducing the burden on healthcare systems.

## Figures and Tables

**Figure 1:**
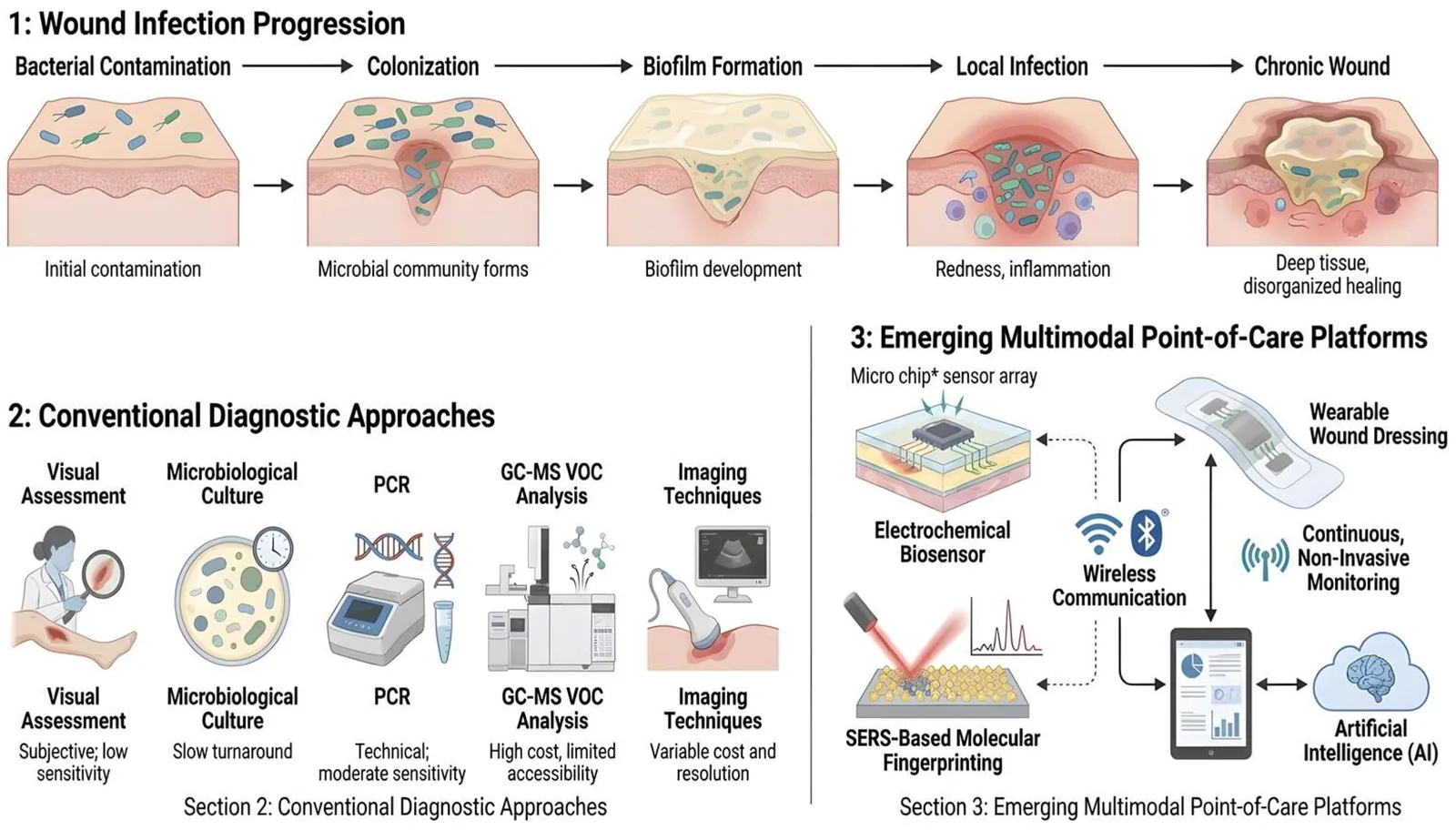
Overview of wound infection progression and emerging diagnostic technologies. The image is created with https://chat.figurelabs.ai (Accessed on 24 September 2026).

**Figure 2:**
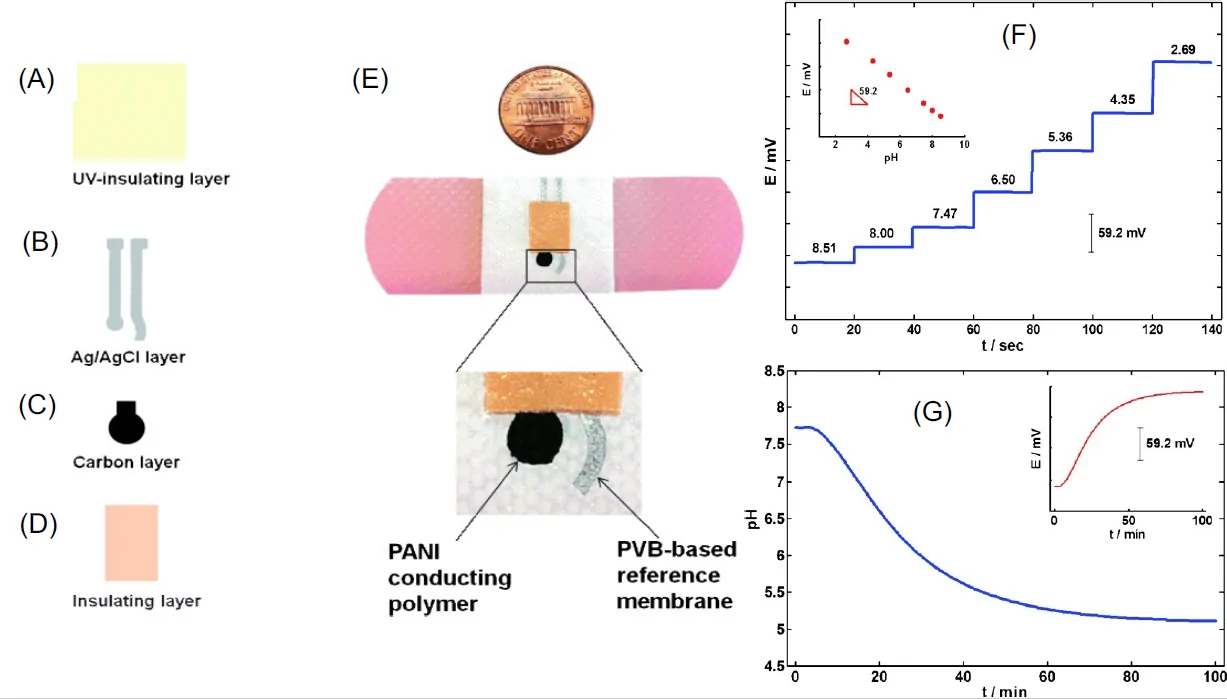
Fabrication and analytical performance of a bandage-based potentiometric pH sensor for wound monitoring. (**A**–**D**) Sequential screen-printing of a transparent UV-cured insulating layer, Ag/AgCl conductive layer, carbon working-electrode layer, and skin-colored insulating layer, respectively. (**E**) Assembled bandage sensor incorporating electropolymerized PANI on the carbon working electrode and a PVB-based solid-state reference membrane. (**F**) Potentiometric response and calibration of the bandage sensor using human-serum-based simulated wound samples with pH values ranging from 7.93 to 4.07. (**G**) Real-time monitoring of pH changes over 100 min using a PEG hydrogel that simulated the wound environment; the inset shows the corresponding electromotive-force response [57]. Reprinted with permission from John Wiley and Sons [57]. Copyright © 2014 John Wiley and Sons. License No. 6354840696564.

**Figure 3:**
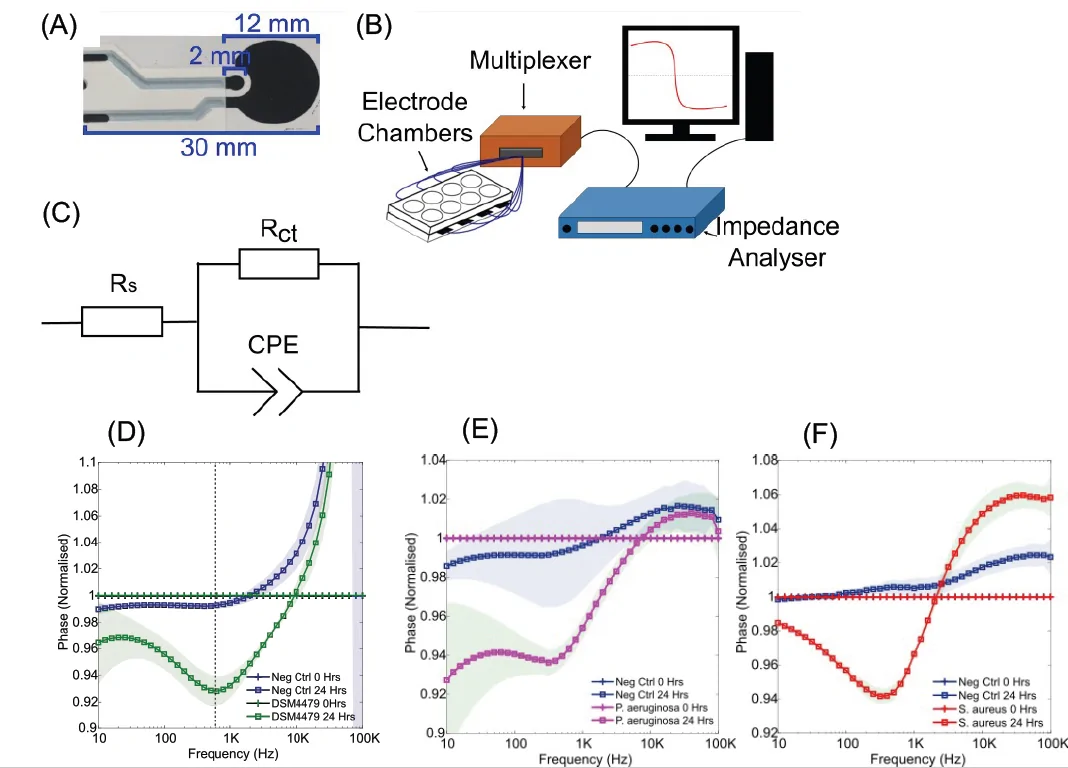
Experimental configuration and normalized phase responses of the impedimetric platform for detecting wound pathogens in simulated wound fluid. (**A**) Disposable screen-printed carbon electrode on a polyethylene terephthalate substrate with the electrode dimensions indicated; (**B**) experimental setup comprising electrode chambers, a multiplexer, an impedance analyzer, and a computer; and (**C**) equivalent circuit used for data fitting, where (R_s_), (R_ct_), and CPE represent solution resistance, charge-transfer resistance, and the constant-phase element, respectively. Normalized phase spectra recorded during 24 h of bacterial growth are shown for (**D**) *P. mirabilis*, (**E**) *P. aeruginosa*, and (**F**) *S. aureus* in simulated wound fluid. The shaded regions represent ±1 standard deviation (*n* = 4). Reproduced from [61] under the terms of the Creative Commons Attribution (CC BY 3.0) license. Copyright © 2021 The Authors.

**Figure 4:**
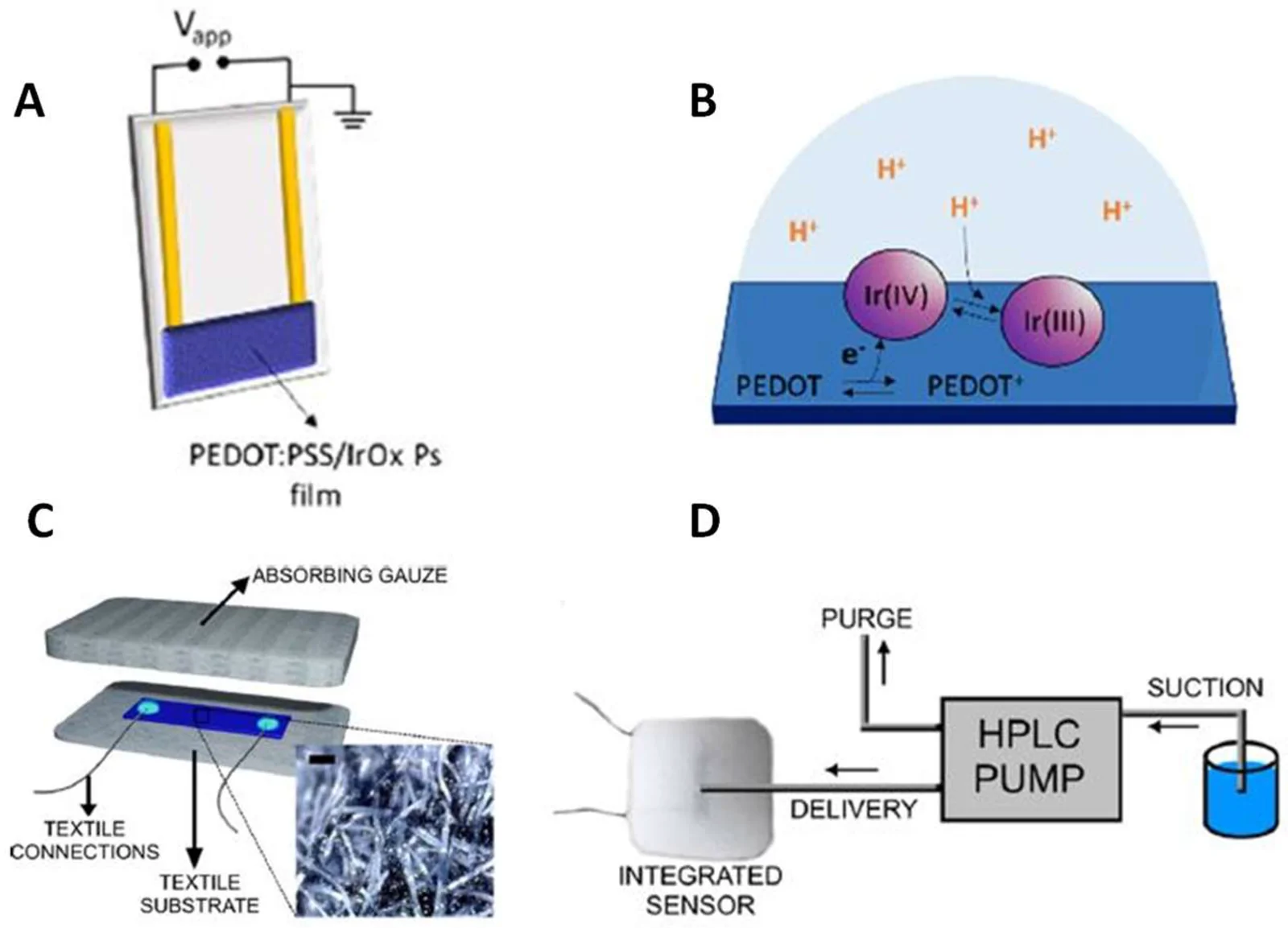
Structure, sensing mechanism, and integration of the reference-free textile pH sensor reported by Mariani et al. (**A**) Two-terminal sensor comprising an IrO_x_ particle-functionalized PEDOT:PSS film patterned between two gold electrodes. (**B**) Proposed spontaneous electrochemical-gating mechanism at the IrO_x_–PEDOT interface, through which pH-dependent charge transfer modulates the hole concentration, conductivity, and measured current of PEDOT. (**C**) Textile smart-dressing architecture incorporating the sensor and an absorbent gauze layer that transports simulated wound exudate to the sensing region. (**D**) Dynamic testing setup employing an HPLC pump to continuously deliver simulated wound exudate with different pH values across the textile-integrated sensor. Reproduced from [73] under the terms of the Creative Commons Attribution 4.0 International (CC BY 4.0) license. Copyright © ACS Sensors (2021).

**Figure 5:**
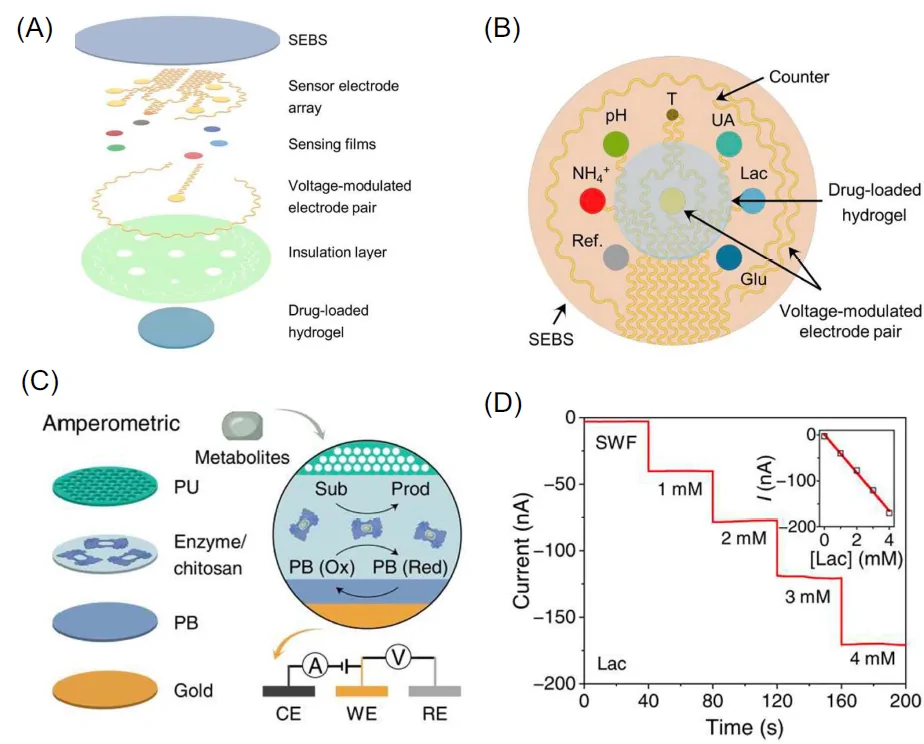
Stretchable wireless bioelectronic patch for multiplexed wound monitoring and treatment. (**A**) Layered configuration of the patch. (**B**) Arrangement of the biomarker sensors and therapeutic electrodes. (**C**) Configuration and detection mechanism of the amperometric enzyme sensors. (**D**) Lactate response and calibration over 0–4 mM in simulated wound fluid. Adapted from SCIENCE ADVANCES [67] under the terms of the Creative Commons Attribution 4.0 International (CC BY 4.0) license Copyright © 2023.

**Figure 6:**
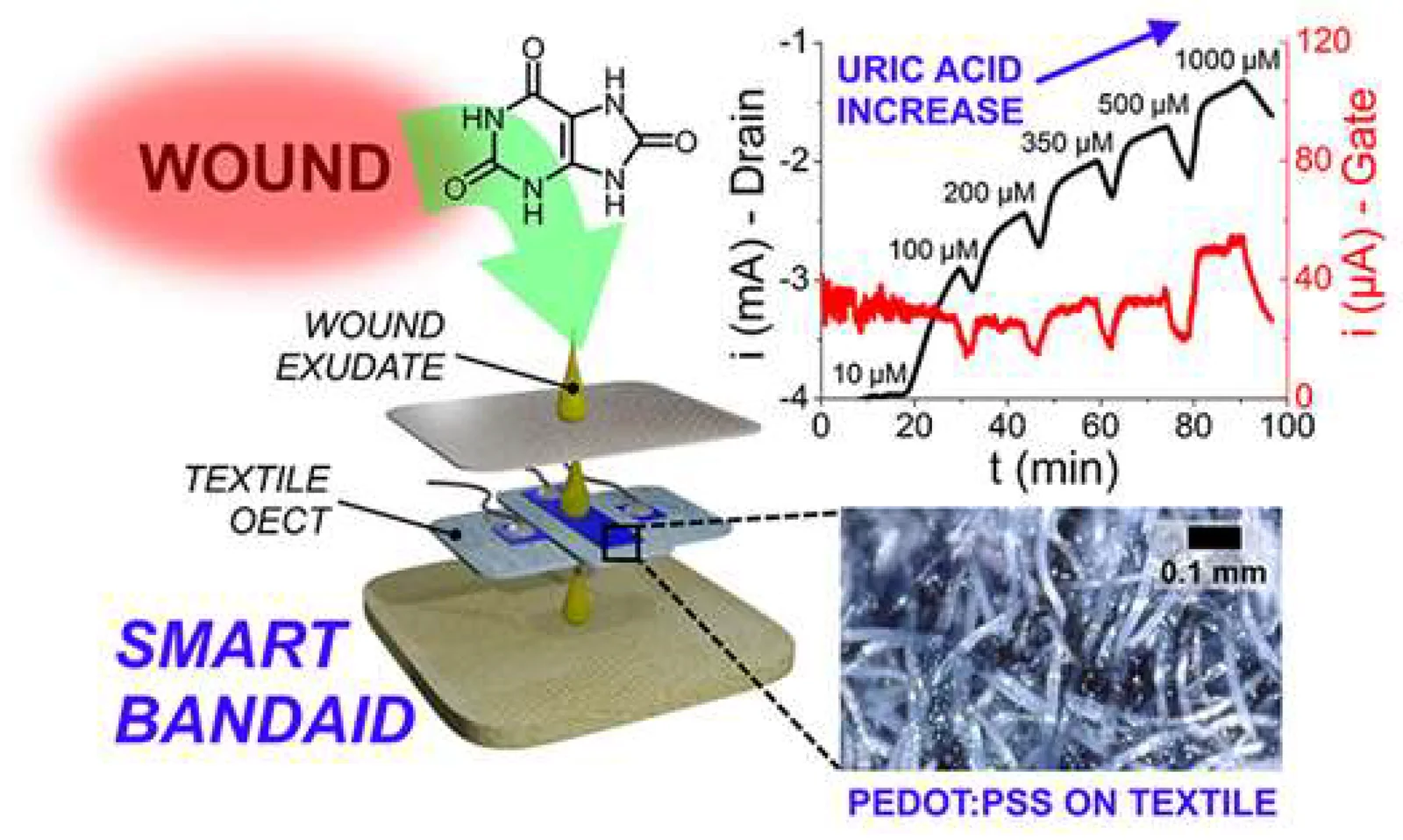
Textile OECT-based smart bandage for uric acid monitoring which illustrates the screen-printed PEDOT:PSS gate and channel patterns on polyester–rayon gauze, the cross-like assembly with intermediate permeable hot-melt adhesive and absorbent layers, and representative calibration plots showing normalized drain current versus uric acid concentration under both static and flow conditions in simulated wound exudate. Reproduced from [80] under the terms of the Creative Commons Attribution 4.0 International (CC BY 4.0) license. Copyright © ACS Sensors (2023).

**Figure 7:**
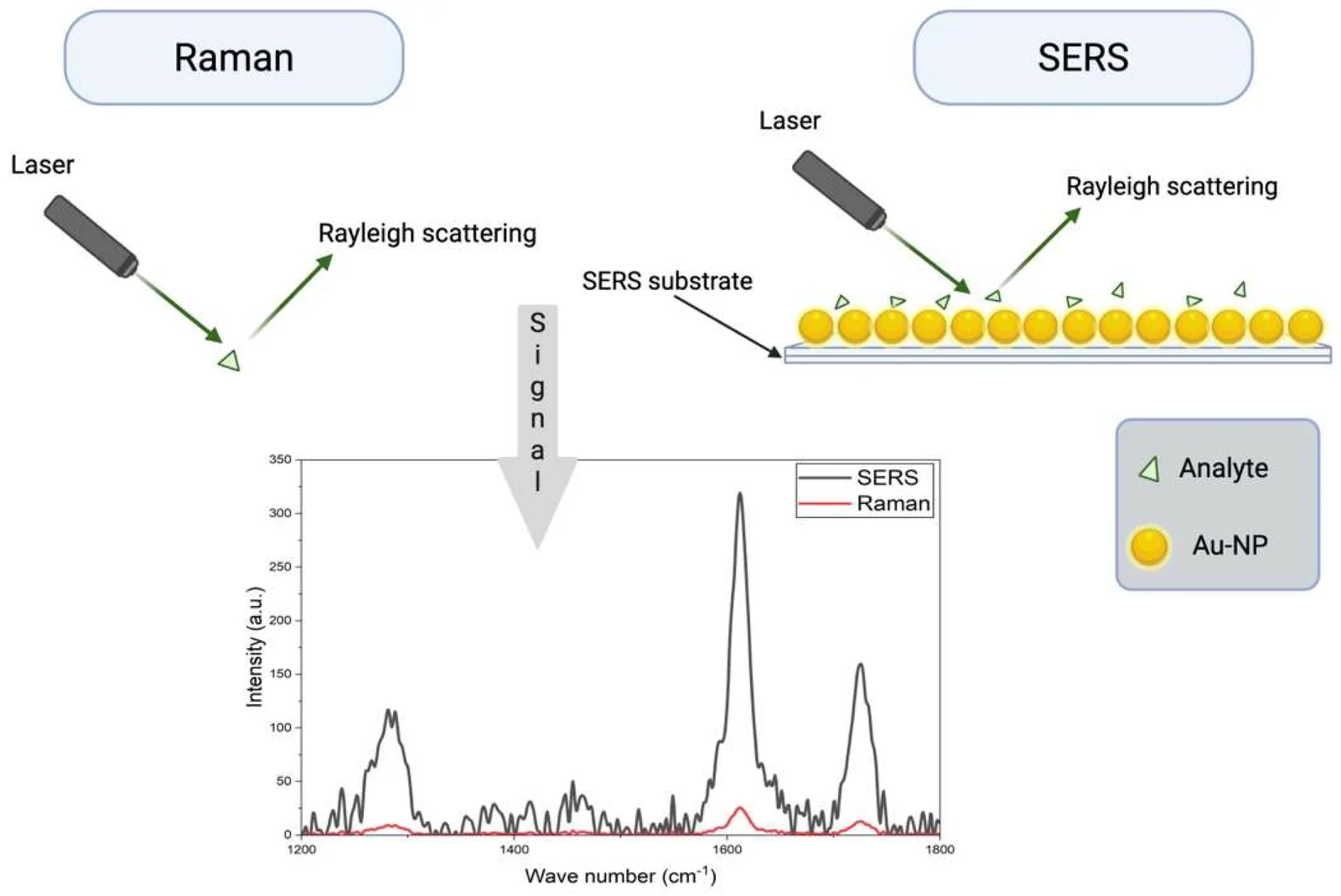
Schematic and spectral comparison of traditional Raman spectroscopy and Surface-Enhanced Raman Scattering (SERS). (**Left**) In conventional Raman spectroscopy, a laser excites the sample producing weak Raman signals alongside Rayleigh scattering. (**Right**) In SERS, analyte molecules interact with gold nanoparticles (Au-NPs), dramatically amplifying the Raman signal via localized surface plasmon resonance. (**Center**) The spectral plot shows the SERS spectrum (black) exhibiting significantly stronger peaks than the traditional Raman spectrum (red). Created in BioRender. Mohamed, R. (2026) https://BioRender.com/hw8lv8o (Accessed on 24 September 2026).

**Figure 8:**
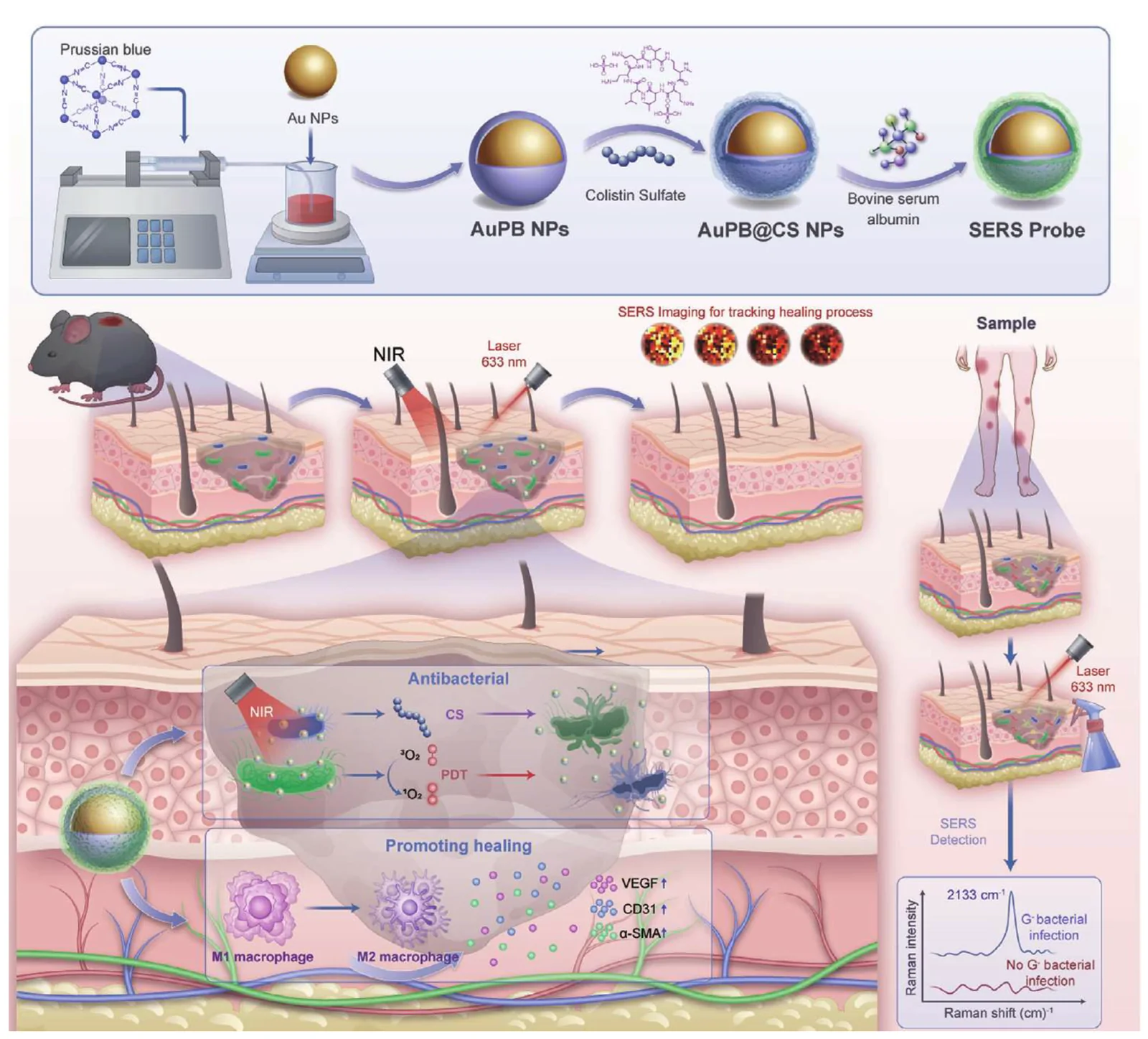
Silent-region SERS nanoplatform for wound infection monitoring and therapy. Au nanoparticles coated with Prussian blue, a Raman reporter at 2133 cm^−1^ and near-infrared photosensitizer, are loaded with colistin sulfate and stabilized with bovine serum albumin. The probe enables background-free SERS tracking of Gram-negative bacterial load in infected mouse wounds during combined photodynamic–antibiotic therapy, and detection of Gram-negative infection in clinical wound samples. Reproduced from [111] under the terms of the Creative Commons Attribution (CC BY) license.

**Figure 9:**
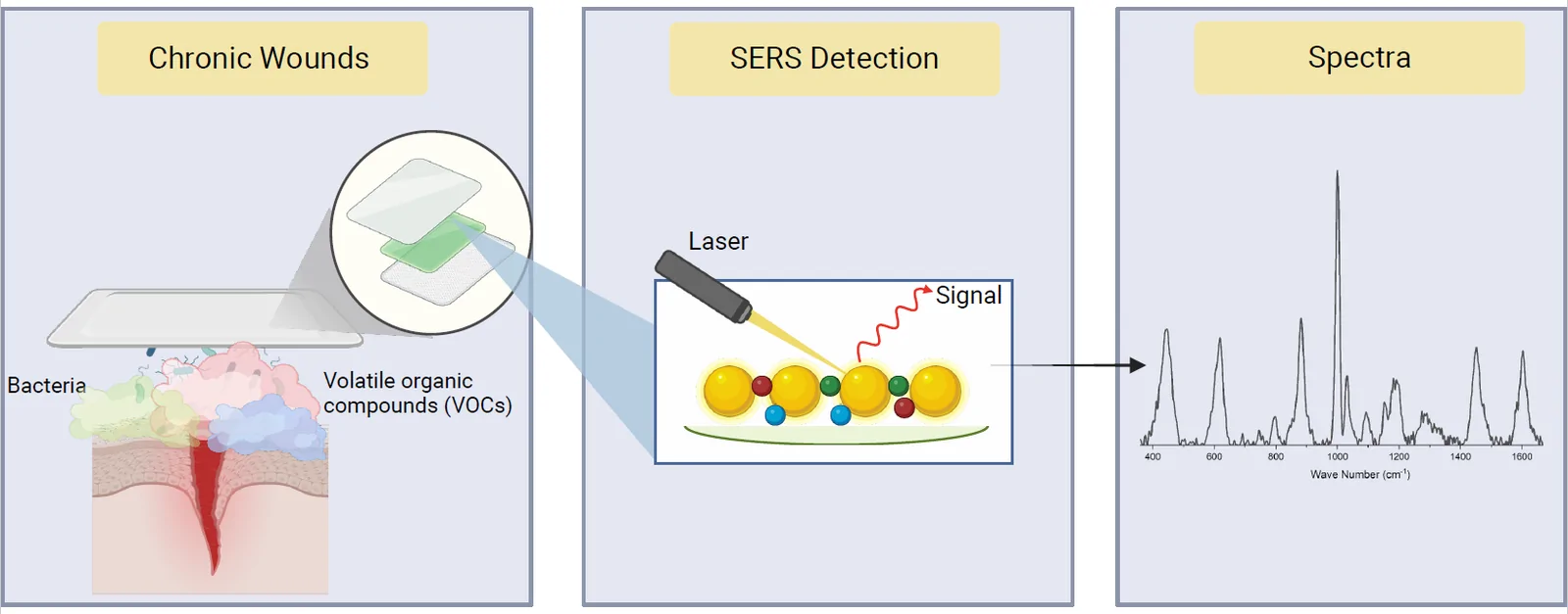
Schematic illustration of VOC-based wound infection detection using SERS. Bacteria present in chronic wounds release volatile organic compounds (VOCs), which are captured and enhanced by plasmonic SERS substrates for spectral analysis and bacterial identification. Created in BioRender. Mohamed, R. (2026) https://BioRender.com/5m60t8f (Accessed on 24 September 2026).

**Figure 10:**
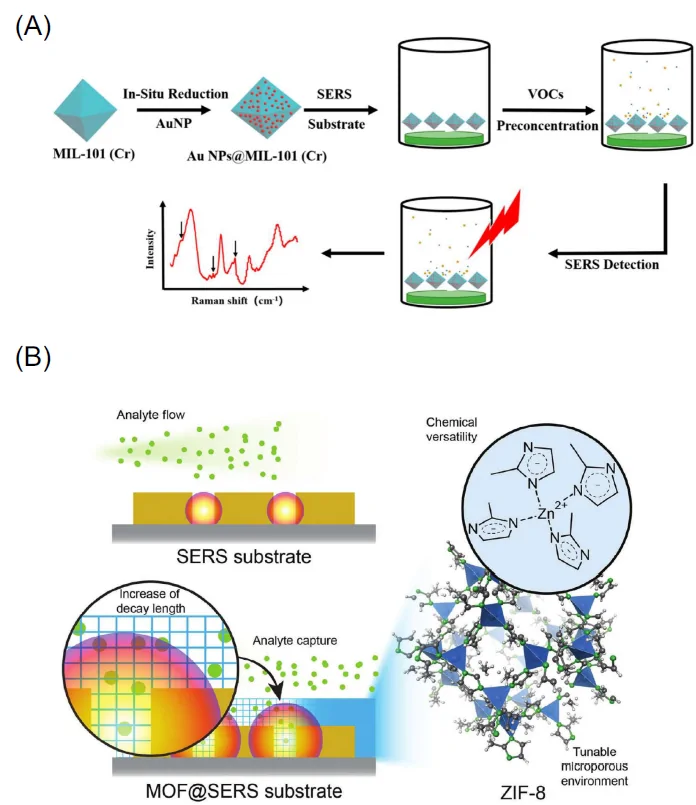
MOF-integrated SERS for VOC detection. (**A**) Workflow of a MOF–SERS VOC sensor: Au nanoparticles grown in situ within MIL-101(Cr) form the SERS substrate, onto which gaseous VOCs are preconcentrated before SERS detection. Reproduced from [127] under the terms of the Creative Commons Attribution (CC BY) license. (**B**) Role of the MOF: compared with a bare SERS substrate, a MOF@SERS substrate captures and accumulates analyte within the plasmonic near-field and increases the field decay length, while offering chemical versatility and a tunable microporous environment (illustrated for ZIF-8). Adapted from [105] (Figure 4a; original panel label removed) under the terms of the CC BY license.

**Figure 11:**
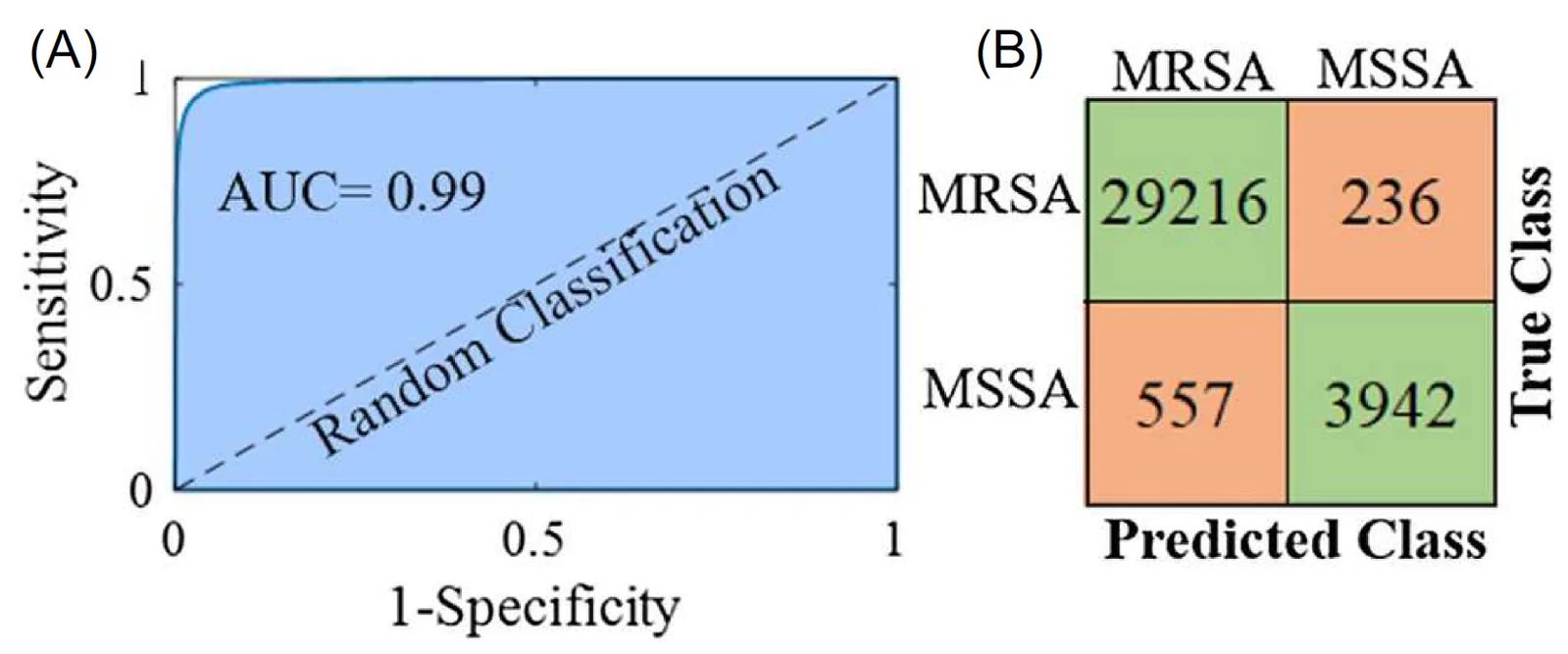
Receiver operating characteristic (ROC) curve (AUC = 0.99) (**A**) and confusion matrix (**B**) demonstrating the performance of a stacked autoencoder-based deep neural network for classifying MRSA and MSSA using label-free SERS spectra [135]. Reproduced from [135] under the terms of the Creative Commons Attribution (CC BY 4.0) license.

**Table 1:** Comparison of electrochemical biosensors for wound monitoring.

Sensing Platform	Electrochemical Technique	Biomarker Type	Analytical Performance	Reference
CSPE/PB/Uricase–BSA–GA/Chitosan	CA	Uric acid	LR: 100–800 µM; Sensitivity: −2.4 nA µM^−1^; 8 h stability: 2.02% RSD	[53]
PET/CUA	SWV	Pyocyanin, uric acid, and nitric oxide	Pyocyanin: LOD 1.0 ± 0.5 µM and LDR 1–250 µM; uric acid: LOD 1.0 ± 0.4 µM and LDR 100–700 µM; nitric oxide: LOD 0.20 ± 0.05 µM and LDR 1–100 µM	[55]
LIG/MoS_x_	SWV	Pyocyanin and phenazine-1-carboxylic acid	Pyocyanin LOD: 0.19 µM in BHI and ≈1.3 µM in WSM; phenazine-1-carboxylic acid LOD: 1.2 µM in BHI	[56]
CSPE/PANi	POT/OCP	pH	Sensitivity: −57.9 ± 0.6 mV pH^−1^ in Human Serum-based samples; continuous monitoring: 100 min in PEG hydrogel	[57]
PET/ITO/PANi	POT/OCP	pH	LR: pH 4–10; Sensitivity: −55 mV pH^−1^; drift: 0.16–0.20 pH units per 24 h; wireless monitoring: 15 h	[58]
PES/PU-AuNP/PANi	POT/OCP	pH	LR: pH 4–10; Sensitivity: 58.9 mV pH^−1^; 14 d stability; 10,000 bending cycles; in vivo monitoring: 21 d	[59]
PET/CSPE	EIS	*Proteus mirabilis*, *Pseudomonas aeruginosa*, and *Staphylococcus aureus*	Detection time in SWF: 2.5 h for *P. mirabilis*, 2 h for *P. aeruginosa*, and 2–5 h for *S. aureus*	[61]

AuNP: gold nanoparticle; BHI: brain–heart infusion; BSA: bovine serum albumin; CA: chronoamperometry; CSPE: carbon screen-printed electrode; CUA: carbon ultramicroelectrode array; EIS: electrochemical impedance spectroscopy; GA: glutaraldehyde; ITO: indium tin oxide; LDR: linear dynamic range; LIG: laser-induced graphene; LOD: limit of detection; LR: linear range; MoS_x_: molybdenum polysulfide; OCP: open-circuit potential; PANi: polyaniline; PB: Prussian blue; PEG: polyethylene glycol; PES: polyethersulfone; PET: polyethylene terephthalate; POT: potentiometry; PU: polyurethane; RSD: relative standard deviation; SWF: simulated wound fluid; SWV: square-wave voltammetry; WSM: wound-simulating medium.

**Table 2:** Wound-associated VOCs: evidence of occurrence in human wounds or tissue, available quantitative reference values, and reported SERS performance.

VOC	Evidence in Human Wounds/Tissue	Quantitative Reference (Matrix)	Reported SERS Detection	Assessment
Dimethyl disulfide	Only VOC reported in 3 of 11 studies [114]; 14/17 infected vs. 0/13 uninfected tissues [116]; severely infected wounds [117]	~10–14 ppbv (maximum medians), culture headspace of *S. aureus* and *P. aeruginosa* [119]	Culture headspace, six species; calibrated in liquid only [121]; quantified above agar cultures [122]	Culture-headspace feasibility shown; gas-phase LOD not established
Hydrogen cyanide	*P. aeruginosa* biofilms, ex vivo human wound model [118]; with dimethyl sulfide, discriminated *P. aeruginosa* biofilms in a collagen wound biofilm model [120]	Quantified by SIFT-MS in a collagen wound biofilm model; compound-specific concentration not reported in the article [120]	Qualitative detection in culture headspace [123]; gas tested at 5 ppm only [124]	Culture-headspace detection demonstrated; ppb-level gas sensitivity not established
2-Butanone	Infrequent but infection-specific in clinical swabs [117]	Up to ~41 ppbv (maximum median), *P. aeruginosa* culture headspace [119]	LOD 4.87 ppm (gas), Au nanostar@MIL-100(Fe) [126]	LOD ~two orders of magnitude above culture-headspace level
Acetophenone	4/17 infected (3 with *P. aeruginosa*) vs. 0/13 uninfected tissues [116]	—	Detected at 1 ppb (gas), TiO_2_NTs/AuNPs@ZIF-8 [125]	Low-ppb gas-phase detection demonstrated; wound concentration unknown
Short/branched-chain fatty acids	Strongest discriminators of infection in a 26-wound pilot study [117]	—	—	Gas-phase SERS performance remains to be established

Absolute wound-headspace concentrations were not reported in the wound studies evaluated here; culture and wound-model values therefore provide approximate context and do not define clinical sensitivity requirements. —, data not identified in the literature evaluated.

**Table 3:** Performance of SERS-based sensors using different substrates for VOC detection.

Strategy	Representative System	Key Advantage	Detected VOC/LOD	Reference
MOF core–shell	AuNPs@MIL-101(Cr)	Selective adsorption, anti-aggregation	Toluene/formaldehyde, 5–75 ppm	[127]
MOF on nanostars	Au nanostars@MIL-100(Fe)	High field enhancement + pre-concentration	Gaseous 2-butanone, eucalyptol, isopropanol (salivary gastric-cancer VOC indicators); 4.87, 3.07, 8.26 ppm	[126]
Au nanocubes@ZIF-8	Au NC@ZIF-8 (10 nm shell)	LSPR enhancement + MOF enrichment; tunable shell (3–40 nm)	Benzaldehyde (lung-cancer breath aldehyde) via 4-ATP capture; reported LOD 2.5 ppb	[128]
Yolk–shell Au@MOF	MesoAu@ZIF-8	Gas capture and enrichment in hierarchical pores	Gaseous benzaldehyde, 0.32 ppb	[131]
3D nanocomposite	TiO_2_NTs/Au@MOF	High surface area, reusable	Acetophenone (gas), detected at 1 ppb; reported LOD 10^−11^ M	[125]

## Data Availability

Data will be available upon request.
